# Bioinformatic Analysis of Patient-Derived ASPS Gene Expressions and ASPL-TFE3 Fusion Transcript Levels Identify Potential Therapeutic Targets

**DOI:** 10.1371/journal.pone.0048023

**Published:** 2012-11-30

**Authors:** David G. Covell, Anders Wallqvist, Susan Kenney, David T. Vistica

**Affiliations:** 1 Developmental Therapeutics Program, National Cancer Institute, Frederick National Laboratory for Cancer Research (FNLCR), Frederick, Maryland, United States of America; 2 Department of Defense Biotechnology High Performance Computing Software Applications Institute, Telemedicine and Advanced Technology Research Center, US Army Medical Research and Materiel Command, Fort Detrick, Maryland, United States of America; Thomas Jefferson University, United States of America

## Abstract

Gene expression data, collected from ASPS tumors of seven different patients and from one immortalized ASPS cell line (ASPS-1), was analyzed jointly with patient ASPL-TFE3 (t(X;17)(p11;q25)) fusion transcript data to identify disease-specific pathways and their component genes. Data analysis of the pooled patient and ASPS-1 gene expression data, using conventional clustering methods, revealed a relatively small set of pathways and genes characterizing the biology of ASPS. These results could be largely recapitulated using only the gene expression data collected from patient tumor samples. The concordance between expression measures derived from ASPS-1 and both pooled and individual patient tumor data provided a rationale for extending the analysis to include patient ASPL-TFE3 fusion transcript data. A novel linear model was exploited to link gene expressions to fusion transcript data and used to identify a small set of ASPS-specific pathways and their gene expression. Cellular pathways that appear aberrantly regulated in response to the t(X;17)(p11;q25) translocation include the cell cycle and cell adhesion. The identification of pathways and gene subsets characteristic of ASPS support current therapeutic strategies that target the FLT1 and MET, while also proposing additional targeting of genes found in pathways involved in the cell cycle (CHK1), cell adhesion (ARHGD1A), cell division (CDC6), control of meiosis (RAD51L3) and mitosis (BIRC5), and chemokine-related protein tyrosine kinase activity (CCL4).

## Introduction

Identifying disease-specific genetic signatures offers the potential for selecting therapeutic targets and discovering small molecules that affect the roles of these targets in cancer cell survival. In practice, disease-specific genetic signatures typically originate from examination of microarray gene expression readouts using diseased cells and applications of *in silico* tools for extracting important (i.e. signature) gene subsets [1]–[3] and pathways (IPA(Ingenuity^R^Systems www.ingenuity.com, GSEA [4], [5] and DAVID [6]). Typically cells used for microarray analysis represent a relatively homogeneous population. Less typical, yet not infrequent, cases involve microarray readouts from patient tumor biopsies [7], [8] where difficulties in assigning disease-specific genetic signatures arise from cellular heterogeneity associated with the tumor/non-tumor composition within each tissue sample. In an effort to analyze genetic readouts from heterogeneous samples and propose potential therapeutic targets, a multi-step analytical methodology is proposed to collectively analyze related, yet different, sets of microarray data. The first step in this methodology identifies disease-specific genes, and their associated biochemical pathways, using pooled microarray readouts from patient tissue biopsies and from an isolated tumor cell. The joint analysis of biopsy and tumor cell microarray readouts offers the opportunity to distinguish the genetic role of genes associated with the tumor microenvironment from that of genes associated with the isolated tumor cell. Genes derived from these readouts, and their associated pathways, serve to identify disease-specific features that are assumed, in part, to affect *in situ* tumor cell survival. The second component of this methodology analyzes the individual (i.e. unpooled) microarray readouts from individual patient tissue biopsies to determine clusters of genes, and their associated pathways, that exhibit consistent expression patterns for these patients. These clustering results serve as a check of the isolated tumor cell and pooled *in situ* derived disease-specific genetic signatures that were found in the first step of the methodology. The third step in this methodology provides a bridge for connecting individual patient gene expression signatures, identified in step two, with patient-derived RT-PCR measures of a putative disease-specific marker. An integral component of this last step is the application of a novel linear model, using microarray signal intensities, to assist in interpreting microarray readouts derived from heterogeneous patient biopsies. The over-arching theme of this methodology is to construct a means to jointly analyze microarray readouts from pooled and individual tissue biopsies, with microarray readouts from an isolated tumor cell, combined with patient-derived RT-PCR measures of a diagnostic marker, to identify a small set of genetic features that collectively span this data and may have potential as therapeutic targets.

ASPS is an exceedingly rare chemo-resistant sarcoma that encompasses less than 1% of all soft tissue sarcomas. Originally described in 1952 [9], this tumor is found primarily in adolescents and young children, is characterized by periods of latency, extremely slow growth and multi-organ metastasis with a partiality to the lung and brain. ASPS is resistant to both radiation and standard chemotherapeutic regimens [10]–[12] and exhibits a non-reciprocal chromosomal translocation, der(17)t(X;17)(p11;q25) [13]. Seminal work by Ladanyi and co-workers [14] indicate that this translocation fuses the C-terminal region of transcription factor TFE3, located at Xp11, to the N-terminal region of the ASPL gene at 17q25. Alternative fusion junctions have been observed and result in expression of two tumor specific fusion transcripts, ASPL-TFE3 type 1 and type 2 and their chimeric proteins, which are thought to function as transcription factors. In an effort to identify new targets for ASPS, in-vivo [15] and in-vitro [16] models of the disease have been recently developed. The xenograft model of ASPS, established in immunocompromised mice, maintains characteristics consistent with the original ASPS tumor including tumor histology, expression of the ASPL-TFE3 type 1 fusion transcript and the ASPL-TFE3 type 1 fusion protein, as well as maintenance of the t(X;17)(p11;q25) translocation characteristic of ASPS. The ASPS xenograft model exhibits stable expression of many up-regulated ASPS gene transcripts including those involved in angiogenesis (ANGPTL2, HIF-1 alpha, MDK, c-MET, VEGF, TIMP-2), cell proliferation (PRL, PCSK1), metastasis (ADAM9) as well as the transcription factor BHLHB3 and the muscle specific transcripts TRIM63 and ITGβ1BP3, and is characterized by the development and maintenance of a functional vascular network, a clinical feature of this highly vascular sarcoma. ASPS-1, the cell line developed from the xenograft tumor [16], also expresses the ASPL-TFE3 type 1 fusion transcript and the ASPL-TFE3 type 1 fusion protein, the t(X;17)(p11;q25) translocation characteristic of ASPS and many of the up-regulated ASPS gene transcripts identified in the xenograft model. ASPS-1 retains the ability to produce highly vascularized ASPS tumors in NOD.SCID/NCr and SCID/NCr mice. These tumors express selected ASPS markers similar to those of the original patient tumors as well as to the xenograft ASPS tumor.

The proposed methodology will be used to identify ASPS-specific genetic signatures from i) patient microarray readouts using tissue biopsy samples of primary and metastatic tumors of alveolar soft part sarcoma (ASPS) [17], ii) an immortalized cell line developed from a lymph node metastasis of one patient [16], and iii) biopsy-derived RT-PCR data from the ASPL-TFE3 fusion transcript used in the diagnosis of this disease. The analytic workflow consists of jointly analyzing microarray data from the pooled patient biopsies and the isolated tumor cell, ASPS-1, and applying Principal Component Analysis (PCA) to determine a subset of genes that characterize this data. This subset of genes is then subjected to two methods of clustering, hierarchical for the ASPS-tissue and ASPS-1 data and Self-Organizing Maps (SOMs) for these genes' expressions across the individual (i.e. non-pooled) patient tissue data. The results of these independently conducted clustering analyses are then examined collectively for the occurrence of genes, and their pathways, that consistently exhibit patterns of over and under expression across this data. Genes, and their pathways, jointly indicated in these parallel analyses are further analyzed by comparisons to the patient-derived RT-PCR measurements of the ASPL-TFE3 fusion transcript. This step uses a novel algebraic model to identify gene subsets, and their associated pathways, that also bear a correlative relationship with patient measures of the ASPL-TFE3 fusion transcript. Patient gene expressions negatively correlated with patient transcript levels represent controls for evaluating the cellular response to ASPS translocation, while patient gene expressions positively correlated with patient transcript levels are proposed as potential therapeutic targets.

Collectively these results identify 75 potential ASPS-specific therapeutic targets that appear in 29 GSEA pathways. The ASPS-specific GSEA pathways are nearly equally divided between cell-cycle related processes, with inclusion of many of the current putative ASPS target genes, including MET and FLT1, and processes related to the tumor stromal microenvironment, with an emphasis on pathways and genes involved in immune surveillance, chemokines and focal adhesion. These results establish a strong interdependency for tumor cell survival on the intrinsic pathways driving cell proliferation and on the stromal environment. One novel component of this interdependency finds a connection between cell-cycle related tyrosine kinase pathways and chemokines involved in controlling their kinase activity; an indication that therapeutic strategies aimed at cell-cycle related genes and microenvironment related genes may be beneficial. Collectively these results support a broader range of potential ASPS-specific therapeutic targets than had been previously considered for the treatment of this chemo-resistant sarcoma.

## Methods

### Tumor Acquisition

ASPS tumors were obtained from surgery, following prior informed written consent, under National Cancer Institute clinical research protocol 05-C-N138, approved by the U.S. National Cancer Institute (NCI) Institutional Review Board (IRB). The NCI IRB, in conjunction with the NCI ethics committee, reviewed the protocol annually and approved all tumor acquisitions and progress of the protocol. The Alliance Against Alveolar Soft Part Sarcoma (TAAASPS) assisted in the acquisition of ASPS tumors utilized in this study. The research protocol followed is in compliance with the Helsinki Declaration of conduct of research using human patients. A detailed description of the patient/tumor characteristics utilized in this present study, including the methodologies for ASPS diagnosis, isolation of RNA, reverse transcription and microarray data acquisition have been previously described [17].

### Quantification of ASPL-TFE3 Fusion Transcripts by Real-Time RT-PCR

SYBR Green chemistry was used to detect primer specific amplicons. Reaction volume (20 µl) included 10 µl Quantitect SYBR Green PCR mastermix (Qiagen, Valencia, CA), in DNase free water (6 µl), 2 ng cDNA (2.5 µl) and 4 µM of forward and reverse primers (1 µl each). The 24 bp forward primer (TTCA GCTA AGTT GCCG AAGT CCCT) corresponds to nt 893–915 in exon 7 of ASPL. The reverse primer (TGAA TCGC CTGC GACG CTCA ATTA) corresponds to nt 1296–1319 in exon 7/8 of TFE3. Reactions were performed in triplicate and universal 18S RNA primers (Ambion, Austin, TX) were used to normalize cDNA amplification. The fluorochrome ROX, included in the PCR mastermix, was used as a passive reference. Reactions were performed using an ABI7500 thermocycler (Applied Biosystems, Step One Plus Real Time PCR System, Foster City, CA). Cycling conditions consist of a single 10 minute/95°C activation step followed by 45 cycles of 95°C/15 seconds, 60°C/60 seconds and 72°C/60 seconds with fluorescence measurements taken in the elongation step of each cycle. Fold changes in expression were calculated manually from Ct values.

### Statistical Analysis

**Figure 1:**
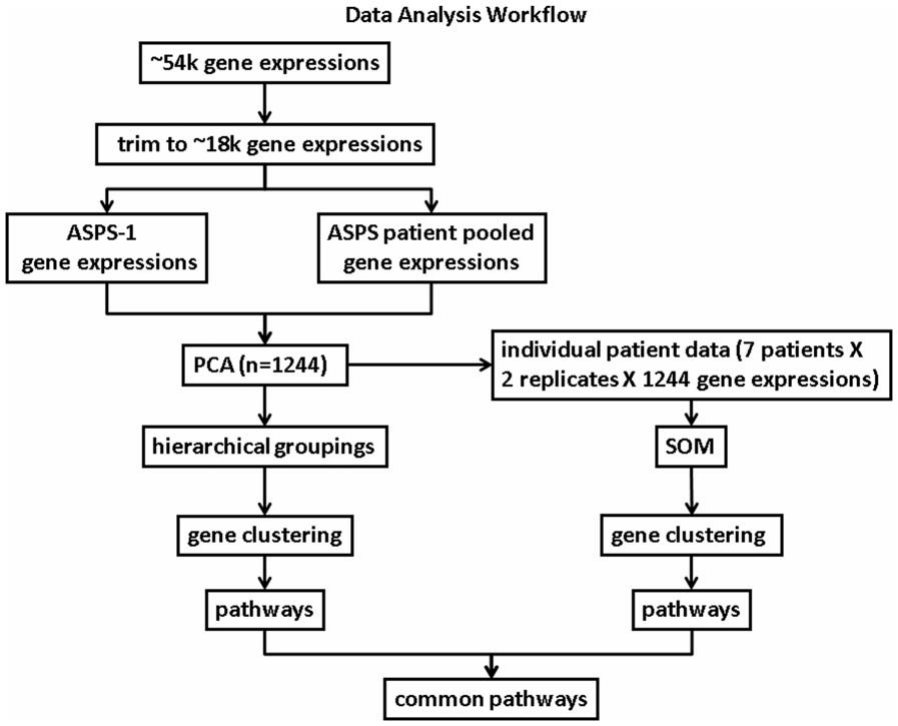
Data Analysis Workflow. The expression of 54,675 genes, done in duplicate, was measured for ASPS tissue biopsies and the ASPS-1 tumor cell. Selecting only present (P) calls trimmed this starting set to 17,698 gene expressions. Next, these ∼18 k candidate genes were analyzed using Principal Component Analysis (PCA). PCA identified 1244 genes that distinguished the pooled patient data from the ASPS-1 tumor cell data. From this point forward these 1244 genes were analyzed, in parallel, for the pooled patient/ASPS-1 tumor cell data (left-most path), and the individual patient gene expressions (right-most path). The left-most path used conventional hierarchical clustering to identify gene clusters. Clustered genes were then used to reveal a set of ASPS-specific pathways. The right-most path analyzed the individual patient measures of these same 1244 gene expressions, using self-organizing maps (SOMs), to cluster genes according to similarities in gene expressions across patient samples. These gene clusters were also analyzed to identify their set of ASPS-specific pathways. The final step in this process selected only pathways and their constituent genes that are shared amongst the ASPS-specific pathways identified from each parallel analyses.

The goal of the analytic workflow, shown schematically in **Figure 1**, is to identify a subset of gene expressions, derived jointly from pooled patient biopsies, individual patient biopsies and an immortalized, ASPS-1, tumor cell, that reveal a consistent picture of ASPS-specific differentially expressed genes and their associated pathways. The strategy first trims the initial set of gene expressions to identify candidate genes that discriminate the pooled patient microarray readouts from the cell-based microarray readouts. Next, an analysis of only these discriminating genes is conducted, in parallel, using the pooled biopsy and cell-based microarray data and, separately, the individual patient microarray data. The following section provides further details of these steps. Additional information appears in the legend to **Figure 1**.

Data trimming of the initial 54,675 mRNA expression measures, done in duplicate, for the patient biopsy and ASPS-1 tumor cell microarray measurements, selected only instances where present (P) calls existed for each gene and its corresponding universal mRNA duplicates. The remaining 17,698 gene expression measures are log transformed, averaged across their replicates and normalized to universal reference RNA, which consisted of a mixture of RNAs from non-tumor adult male and female human tissues. Data processing on this trimmed dataset is completed in two steps. First, patient data is pooled to yield an average gene expression for these ∼18 k genes derived from ASPS tissue samples. This data is compared to the corresponding gene expression measures derived from ASPS-1. The pooled patient ASPS gene expression data is referred to hereafter as ASPS-tissue samples, while the cell derived dataset is referred to, as noted earlier, as ASPS-1.

**Figure 2:**
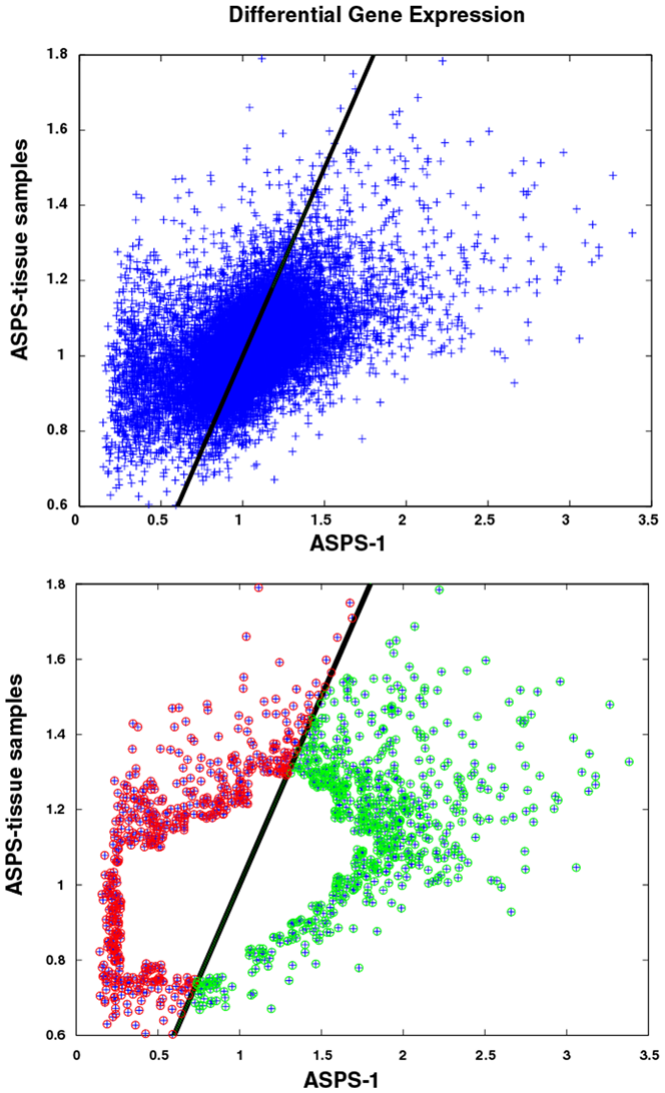
Top Panel: Scatter plot of pooled ASPS-tissue (y-axis) versus ASPS-1 (x-axis) differential gene expression measurements. Data trimming (see Methods: Statistical Analysis) reduced the original 54,798 measurements to 17,698 differentially expressed genes. The diagonal line represents the first principal component (1^st^ PC) from PCA analysis and accounts for 91% of the variation in this data set. The lower panel displays the 1244 gene expressions not associated with the 1^st^ PC. Points in red and green, respectively, correspond to differential expressions relatively higher in the pooled ASPS-tissue versus ASPS-1 gene expressions, and vice-versa. Consistent with the PCA analysis, the 1^st^ PC exactly bisects the pooled ASPS-tissue versus ASPS-1 datasets.

A plot of the ∼18 k gene expressions derived from pooled ASPS-tissue (y-axis) versus ASPS-1 gene expressions (x-axis) appears in the top panel of **Figure 2**. Over and under expressed genes within the pooled ASPS-tissue samples are assumed to reflect aggregate measures of gene expression from normal and tumor cells, while extremes in gene expressions from the ASPS-1 sample are assumed to represent only the tumor subpopulation. The inherent overlap of tumor cells in both populations is consistent with the correlated nature of their gene expressions, as evidenced by the strong band running diagonally across the image in the top panel of **Figure 2**. Each subpopulation is comprised of genes over and under expressed, relative to universal mRNA and, more importantly, relative to each other. Principal Component Analysis (PCA) is a statistical procedure that converts a set of correlated variables into sets of uncorrelated variables, called principal components (PCs). The number of principal components is less than or equal to the number of original variables, which for the dataset used here consists of two variables; gene expressions derived from ASPS-tissue samples and ASPS-1. This transformation assigns the 1^st^ PC to data associated with the highest variation, with each succeeding PC having the highest variance possible under the constraint that it is not correlated with the preceding PCs. The 1^st^ PC for this dataset is displayed as the dark line running diagonally in the top panel of **Figure 2**. This line passes through the majority of the data, and reflects the highly correlated (i.e. expected) nature of this dataset. Standard PCA analysis determines the fraction of the variance associated with each PC. For the data displayed here, 91% of the variation is associated with the 1^st^ PC; leaving the remaining 9% of variation *not* accounted for by the correlation implicit in this data set. The 2^nd^ PC runs perpendicular to the 1^st^ PC. Data associated with the 1^st^ PC are assumed, herein, to represent background genes with proportional expressions across both the pooled ASPS-tissue and ASPS-1 samples. As such, these genes represent cases where the pooled ASPS-tissue and ASPS-1 expressions are not different, and thus less interesting. Genes with differences in expressions between these samples are assumed to be associated with the 2^nd^ PC. These genes are selected by eliminating genes within the most densely populated regions flanking the 1^st^ PC. A grid-based approach was used to determine the number of nearest neighbors within each cell of the grid. Cells with neighbor counts above the 90^th^ percentile of counts for all grid cells were eliminated, to yield the 1244 genes with differential expressions that were not associated with the 1^st^ PC (see lower panel **Figure 2**). These remaining differentially expressed genes can be further stratified according to their relative differential expression in pooled ASPS-tissue versus ASPS-1. These gene expressions reflect differences resulting from samples derived from ASPS-1 versus samples derived from pooled patient samples. The lower panel in **Figure 2** displays, in red, genes over expressed in pooled ASPS-tissue relative to ASPS-1, and in green genes relatively over expressed in ASPS-1 versus pooled ASPS-tissue samples.

### Pathway Analysis

The 1244 differential genes associated with the remaining 9% of variation are analyzed using Gene Set Enrichment Analysis (GSEA). This publically available tool (http://www.broadinstitute.org/gsea/) calculates a statistical probability for the likelihood of gene pairs (or higher) occurring randomly within a set of curated pathway annotations [5], [18]. The GSEA results for gene subsets is reported in tabular form to include the HUGO name, the identity of the curated pathway annotation, brief descriptions of each pathway and the statistical significance of this finding. In lieu of reporting GSEA results in tabular form, abridged pathway descriptions will appear in the text. All pathways reported in this analysis achieve a statistical significance below 0.05. The complete set of GSEA results appear in **Figures S1** and **S2**.

### SOM Clustering

The differential gene expressions for the filtered genes for each patient sample consists of 1244 gene expressions for 7 patients done in replicate (1244×7×2). Each record represents the signal associated with a gene's expression as measured in the ASPS tissue samples. Pooling of this dataset was used above to derive the subset of trimmed genes displayed in the lower panel of **Figure 2**. The SOM analysis [19] examines the individual (i.e. non-pooled) patient data for the existence of gene expression *patterns* shared across all patient samples. The underlying assumption is that each tissue sample's tumor/non-tumor heterogeneity is preserved, thus fixing the relative differential gene expressions in that sample. In other words, if a patient's ASPS tissue consists of 30% tumor and 70% non-tumor, these fractions apply to all gene measurements for that patient. Consequently the extent of heterogeneity remains constant within each patient's tissue sample. Based on this assumption, the profile of each gene's differential expression across all patients represents an ASPS-specific tissue signature for the seven patients analyzed here. Gene clusters found from the PCA analysis of pooled ASPS-tissue and individual ASPS-tissue expressions that are also found in the SOM clustering of individual patient gene expressions are indicative of a consistent set of disease-specific pathways and genes. The existence of a consistent set of genes provided reciprocal support for their inclusion as disease-specific genes. Stated differently, gene clusters identified from the PCA-derived subsets of pooled patient tissue samples and ASPS-1 should also cluster on the basis of similarities in gene expression patterns derived from individual patient biopsies. This type of cross checking provides an internal consistency check for identifying ASPS-specific genes and their pathways.

### Strategy for Assessing Gene Expression Variations in Tissue Samples from Different Patients

Many cancers, including ASPS, are characterized by chromosomal translocations, resulting in genetic defects involving expression of a fusion gene and protein that can be recognized as a tumor marker. As such, the expression of the fusion protein is a characteristic property of the tumor, with non-tumor cells not expressing this protein. In the consideration of tumor samples from many patients, as well as from different tumor locations on the same patient, the genetic signature of such samples, as measured by gene expression from microarrays, can and will be quite varied for many different reasons. As noted earlier, excised or biopsied tissue samples from tumors contain both tumor cells and non-tumor cells. Further confounding the problem is that expression of any particular gene can be different in the non-tumor tissue versus tumor tissue. Both the amount of tumor and the gene expressions are *a priori* unknown quantities. The strategy proposed here uses a linear, algebraic model to extract, from patient microarray data, gene expression patterns that are representative of the underlying genetic defect.

The strategy assumes that only one tumor type exists within a patient's tissue sample. Microarray analysis assumes that the raw channel intensity signal, I_i,k_, for a particular patient tumor, i, and gene k, is proportional to the total amount of mRNA in the patient's tissue sample, which can be formally written as:
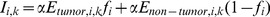
(1)where α is the constant that converts the amount of RNA to a detectable signal, E*_tumor,i,k_* is the RNA expression of gene *k* in patient *i*'s tumor cell portion of the sample, *f_i_* is the fraction of the sample that contained tumor cells, and E*_non-tumor,i,k_* is the RNA expression of the same gene *k* in patient i's non-tumor cell portion of the sample.

Measuring the expression of a reference RNA sample for each microarray, done simultaneously on each microarray, the total signal intensity, *I_ref,k_*, stemming from the RNA expression of gene *k* in the reference sample can be expressed as:
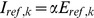
(2)Where α is the constant that converts the amount of mRNA to a detectable signal, as above, and *E_ref,k_* is the mRNA expression of gene *k* in the reference sample.

The gene expression ratio, defined as:
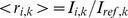
(3)measures the expression of gene *k* in the tumor sample relative to the reference sample. The expression ratio *r_i,k_*, for gene *k* of patient *i*, as referenced to the non-tumor cells of the same sample is:
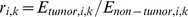
(4)which is not necessarily the same as the <*r_i,k_*>. To illustrate this potential difficulty, consider the case where the reference mRNA, *E_ref_*, is the same as the mRNA derived from non-tumor cells in the patient sample, *E_non-tumor_*. The two expression ratios can now be expressed as a function of the fraction of tumor cells in the patient sample:
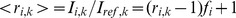
(5)This relationship finds that the two expression ratios <*r_i,k_*> and *r_i,k_* are identical only if *f_i_* is 1, i.e. if the entire excised tumor sample contains nothing but tumor cells. If *f_i_* is different from 1 the two ratios deviate from each other, e.g. for a *f_i_* of 0.1 and a measured <*r_i,k_*> of 10, *r_i,k_* is (10−1)/0.1+1 = 91. Consequently, a sample from a patient's tumor with varying *f* finds the measured ratios to vary dramatically even though the expression of the gene is the same.

### Application of Quantitative RT-PCR to the Known Tumor Signature Gene

When a known genetic defect causes a marker fusion gene to be expressed, different tumor samples can be characterized by performing quantitative RT-PCR on the fusion gene transcript using oligo-primers that span the fusion site. The amount of fusion transcript varies for each tumor, but can be referenced to a specific, but arbitrary patient. As is done with gene expressions, the observable fusion amount, *R_i_*, is related to the tumor fraction in the sample. Assuming that the measure of fusion transcript in the tumor portion of the tissue sample is the same across all tumor cells, the fusion fraction can be written as
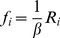
(6)where 1/*β* is the proportionality constant that relates the observed normalized RT-PCR values to the absolute fraction in the sample. The properties of the expression system can be examined by graphing the single channel observables *I_i,k_* and *R_i_* versus each other, i.e.
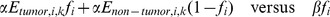
(7)Using the expression ratio *r_ik_*, for gene *k* of patient *i*, as defined above, the left side of the definition of intensity can be expressed as a linear equation in *f_i_*:
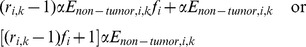
(8)Since all constants in equation (9) are positive, the important part of this linear equation is the slope, and in particular the quantity that determines its sign, (r_i,k_−1). When a graph of gene fusion transcript versus gene expression across all tumor types exhibits a positive slope, then
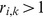
(9)i.e. the expression ratio of the gene is enhanced in the tumor tissue, and likewise if the slope is negative, the gene is expressed less in the tumor tissue. In practice the linear equation *I_i,k_(R_i_)* = *α_k_*+*β_k_R_i_* is evaluated, where *α_k_*, *β_k_* are constant for each gene and independent of the tumor sample. The actual constants determining the proportions and conversions do not need to be evaluated. For purposes of the study conducted here, the analysis simply reduces to identifying instances of a non-zero slope between patient gene expressions and ASPL-TFE3 fusion transcript levels. This is equivalent to identifying patient gene expressions that are positively or negatively correlated with the patient's fusion transcript levels. This observation is rather remarkable in that the analysis avoids the issue of how much of the tissue sample originates from tumor cells. If the observed gene expression data originates from two or more different tumor cell populations the analysis could be amended to take this into account. The method, however, is not suitable to estimate quantitative ratios, as the experimental variations are not cancelled out using standard ratio techniques. However, it is a powerful qualitative method, when the goal is to identify important gene subsets. Gene expressions determined from this approach could be verified with an independent experiment such as quantitative RT-PCR, although a quantitative measure may also be inherently difficult due to variations of the tumor-cell content in sampled tissue specimens. An integral component of this analysis is relating key component of this.

The importance of this result in the context of identifying ASPS-specific genes and associated pathways has profound implications for the methodology proposed here. Specifically, correlative measures of differential gene expressions across patient samples versus RT-PCR measures of ASPL-TFE3 fusion transcript in these same patient samples, represents a valid means of assessing ASPS-important gene subsets and their associated pathways. An additionally important component of this analysis is that it provides a means to integrate the earlier-derived gene cluster results, based on pooled tissue and ASPS-1 gene expressions, with the RT-PCR data. If the disease-specific genes derived from each part of the analysis were not consistent, there would be no basis for extending the results into genes with expressions correlated with the TFE3-ASPL transcript levels.

## Results

The results are presented sequentially, beginning with the clustering results for the pooled patient ASPS-tissue versus ASPS-1 trimmed gene sets (n = 1244), followed by the clustering results of expressions for these same 1244 genes using the individual (i.e. non-pooled) patient tissue samples. Reporting consists of providing GSEA pathway results for clustered gene subsets and collectively integrating these results to identify common over and under expressed genes and pathways. The guiding principle is to use each data mining approach to converge on a set of ASPS-specific signature genes and their associated pathways.

### GSEA Results for pooled ASPS-tissue and ASPS-1 genes

The pooled ASPS-tissue versus ASPS-1 PCA divided the 1244 gene expressions (i.e. trimmed dataset) into 542 genes lying above and 702 genes lying below the 1^st^ PC (see **Figure 2**). Genes above the 1^st^ PC represent relatively greater gene expression in ASPS-tissue samples when compared to ASPS-1. GSEA results for the topmost scoring GO, KEGG or Biocarta pathways for genes over expressed in the ASPS-tissue partition are dominated by pathways associated with the extracellular region;

- proteinaceous extracellular matrix; GO:0005578.
- extracellular matrix; GO:0031012.
- extracellular region and extracellular region part; GO:0044421 and GO:0005576.
  and pathways involved in cellular maintenance and development;
- protease inhibitor activity; GO:0030414.
- cellular morphogenesis during differentiation; GO:0000904.

Together these GSEA pathways point to genes that are involved in maintaining a stable cellular environment. The complete set of GSEA pathways and their 45 pathway genes from these 542 genes are displayed in **Figure S1**.

The GSEA results for the genes having expression greater in ASPS-1 versus ASPS-tissue samples (e.g. below the 1^st^ PC in **Figure 2**) identify pathways associated with;

- cell cycle phase; GO:0022403.
- cell cycle; KEGG cell cycle
- meiotic cell cycle; GO:0051321l.
- m_phase; GO:0000279.
- meiotic recombination; GO:0007131.
- meiosis_I; GO:0007127.
- cell cycle checkpoint; GO:0000075.
- DNA recombination; GO:0006310.
- homologous recombination; KEGG Homologous recombination
- p53 signaling pathway; KEGG p53 signaling pathway

These genetic signals point to pathways that support cellular maintenance and growth. The complete set of GSEA pathways and their 24 pathway genes derived from these 702 genes are listed in **Figure S2**.

### GSEA Results for clustered subsets of pooled ASPS-tissue and ASPS-1 genes

**Figure 3:**
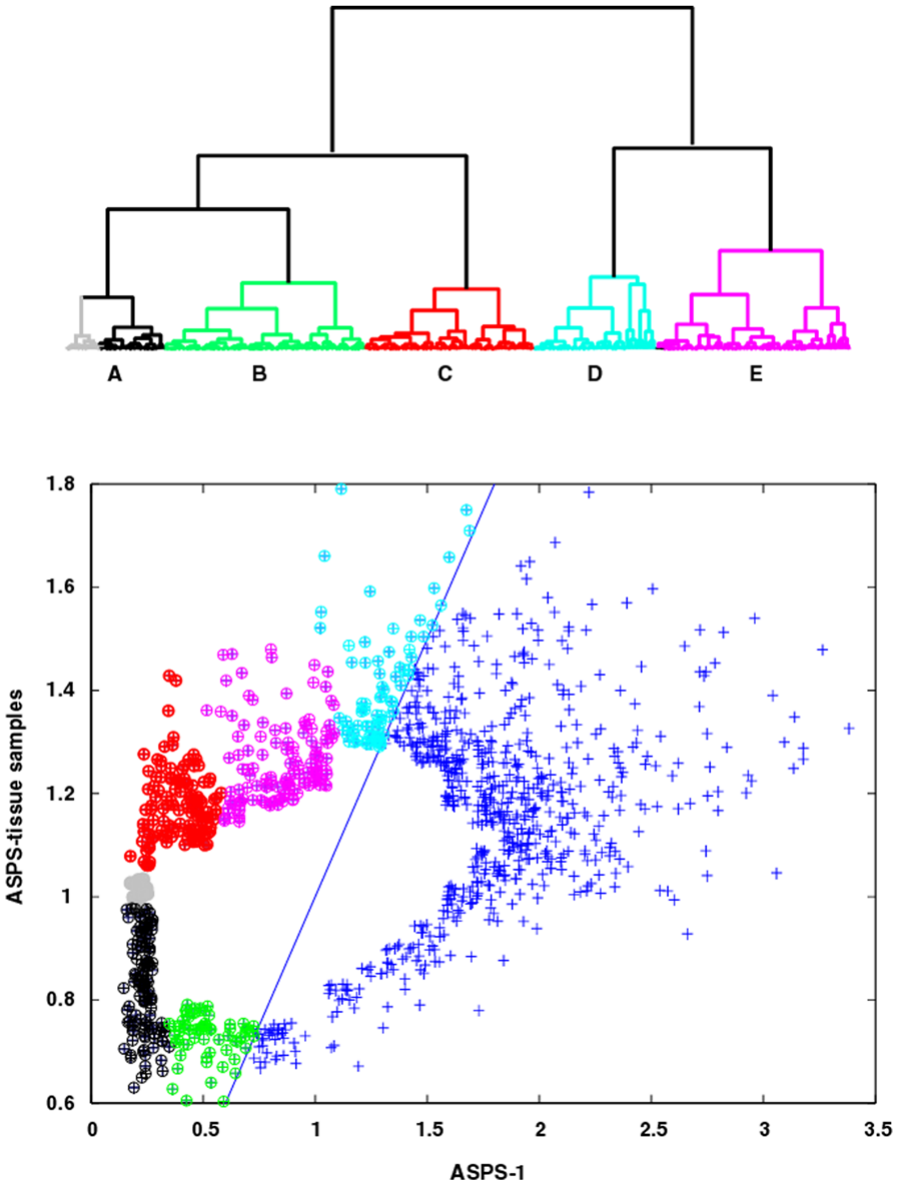
Top Panel; Dendrogram for pooled ASPS-tissue Gene Expressions. Clades are colored to identify samples associated with five meta-clades (DEND meta-clades A, B, C, D and E). The members of DEND meta-clade A with ASPS-tissue values higher than universal RNA are displayed in gray. All other meta-clades are comprised of genes with expression values above universal RNA (i.e. values above 1.0 on the y-axis). Bottom Panel, ASPS-tissue versus ASPS-1 scatter plot where cluster memberships are color-coded to match the meta-clade dendrogram displayed in top panel.

The results in the two preceding paragraphs provide a coarse picture of the GSEA pathways and their associated genes identified from a global assessment of the 1244 genes in pooled ASPS-tissue and ASPS-1 that are not associated with the 1^st^ PC. To obtain a more refined perspective of ASPS-specific pathways, simple hierarchical clustering can be used to identify gene subsets within this dataset. **Figure 3** (upper panel) displays the dendrogram (Euclidian distance metric and Wards clustering) for the pooled ASPS-tissue samples. Clustering divides the 542 genes in the complete ASPS-tissue set into two groups, comprised of three and two sub-clades, respectively (see **Figure 3**, lower panel, for sub-clade members). The GSEA pathways and their associated genes for all dendrogram meta-clades (shortened hereafter to DEND meta-clade to distinguish these results from the SOM results to follow where meta-clades will be referred to as SOM meta-clades) are listed in **Table S1**. DEND meta-clade A, colored **black** in **Figure 3** (lower panel), has GSEA pathways comprised of the extracellular region, Biocarta ASHO and KEGG viral myocarditis. Here hemoglobin (HBA1, HBA2), myosin (MYH11) and collagen (COL4A5, COL6A3) related pathway genes would be expected from tissue-derived samples. The clade associated with the color green (DEND meta-clade B) identifies GSEA pathways for enzyme inhibitory and regulatory activity, membrane and cell fractions and cytosol. These pathways are also consistent with samples taken from ASPS tissue. Noteworthy in this and the previous set of genes (DEND meta-clade A) is that nearly all of these pathway genes are relatively under expressed when compared to universal RNA (i.e. universal RNA normalized expression values below 1.0). Despite the fact that these genes are relatively under expressed compared to universal RNA, there normalized expressions are greater than observed for the ASPS-tissue samples, as these measures should be largely devoid of these genes. The clade associated with the color red (DEND meta-clade C) identifies the GSEA pathways: positive regulation of cell proliferation, extracellular region, chemokine activity, immune response, inflammatory response and defense response. Noteworthy in their sets of pathway genes is the appearance of thrombospondin and various chemokines, whose presence has been shown to inhibit neovascularization and suppress tumor growth in breast cancer xenografts [20]. The genes associated with the color cyan (DEND meta-clade D) identify GSEA pathways: sarcomere, myofibril, contractile fiber part and structural constituent of muscle (genes TTN and NEB), and heparin binding, carbohydrate binding and glycosaminoglycan binding (genes POSTN and MDK). Genes associated with the color magenta (DEND meta-clade E) identify GSEA pathways for focal adhesion and basolateral membrane, which include genes from the integrin family, vascular endothelial growth factors and membrane proteins. The gene subsets for GSEA pathways associated with these 3 latter DEND meta-clades (C, D and E) have expressions above their universal mRNA reference (i.e. normalized expressions greater than 1.0). Furthermore, the genes associated with DEND meta-clade D represent the most over expressed genes in this set; a result consistent with previous studies [17].

**Figure 4:**
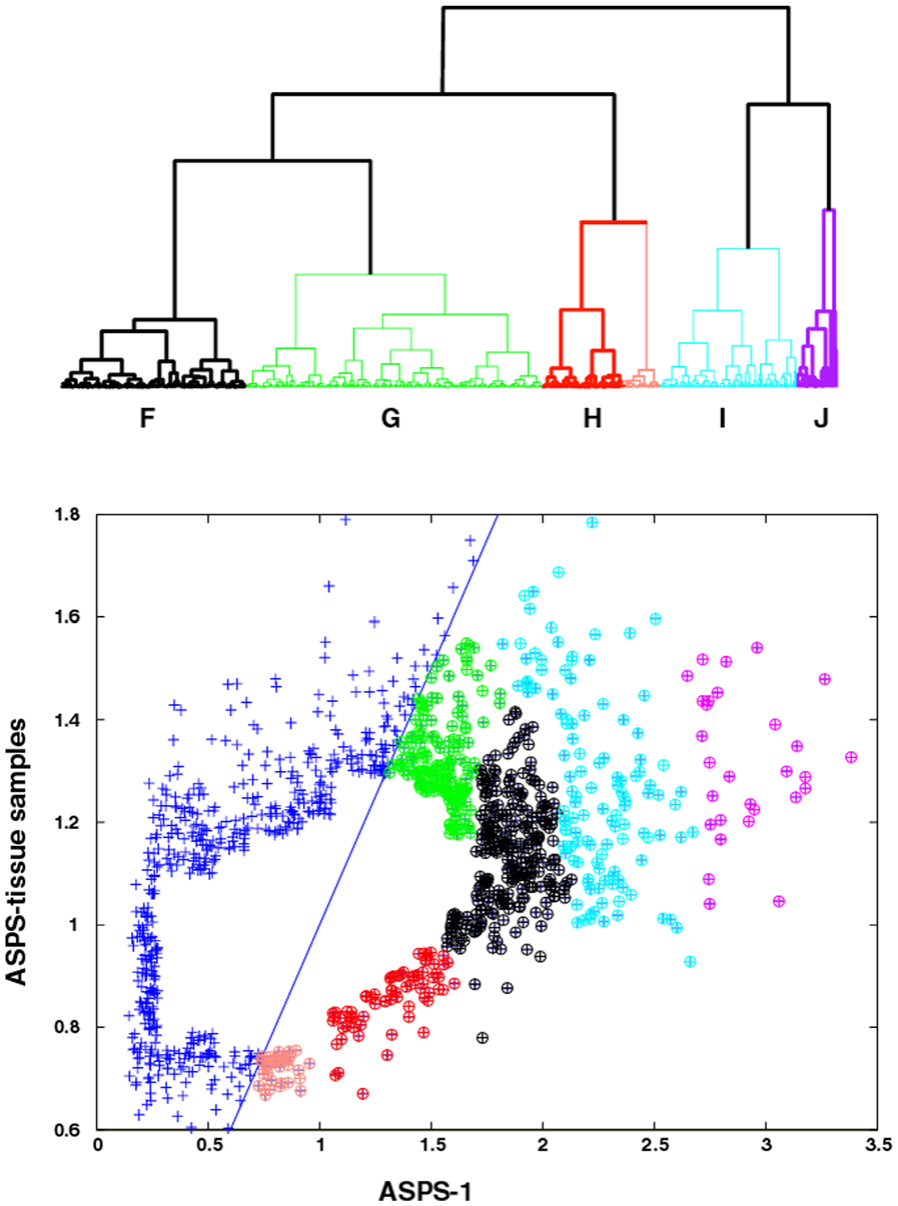
Top Panel, dendrogram for ASPS-1 gene expression. Clades are colored to identify samples associated with five meta-clade memberships (DEND meta-clades F-J). DEND meta-clade members with gene expressions lower than their universal RNA are highlighted in pink. Bottom Panel, ASPS-tissue versus ASPS-1 scatter plot where meta-clade memberships are color-coded to match the dendrogram displayed in the top panel.

**Figure 4** (upper panel) displays the dendrogram (Euclidian distance metric and Wards clustering) for clustering of ASPS-1 gene expressions. Clustering, on the basis of expression levels, divides this gene set into three groups, each comprised of two sub-clades (see **Figure 4**, lower panel, for sub-clade members). The clade associated with the color red (DEND meta-clade H) represents a special case, where the sub-clade colored in pink represents the portion of this gene subset that has expression values below universal RNA. All other meta-clades have gene expressions above universal RNA (i.e. greater than 1.0 on the x-axis). GSEA pathway results for all genes associated with meta-clade H are listed in **Table 1**. These comprise numerous pathways associated with cellular growth. The format of **Table 1** includes GSEA results for the probability of the occurrence of two or more genes within a pathway and the numbers of genes within these pathways. This example serves to illustrate the relatively high significance (compared to the standard of p = 0.05) for these events not representing random occurrences. The over expressed ASPS-1 genes in these pathways consist of BUB1, TTK, BIRC5, CDC23, KIF2C and RAD52, none of which appear in the pink sub-clade of genes under expressed with respect to universal RNA. These genes and their associated pathways represent cellular signals that are both over expressed relative to universal RNA and these expressions are greater than found in the ASPS-tissue samples.

**Table 1:** GSEA results for DEND meta-clade H (red∶pink) for ASPS-1 over expressed genes relative to ASPS-tissue.

Name	ID	# genes	P-val
red∶pink DEND meta-clade H			
Regulation of mitosis	GO:0007088	4	3.88E-04
M phase	GO:0000279	6	4.23E-04
Mitosis	GO:0007067	5	6.85E-04
M phase of mitotic cell cycle	GO:0000087	5	7.65E-04
Cell cycle phase	GO:0022403	6	3.42E-03
Spindle	GO:0005819.	3	4.02E-03
Regulation of cell cycle	GO:0051726	6	4.42E-03
Cell cycle	GO:0007049	8	5.12E-03
KEGG cell cycle	Cell cycle	5	5.14E-03
Cell cycle process	GO:0022402	6	5.91E-03

Column 1 provides a short description of the pathway, column 2 the pathway identifier, column 3 the number of genes from ASPS-1 in this sub-clade that occurs within each pathway, column 4 the statistical significance of this occurrence.

The clade associated with the color **black** (DEND meta-clade F, **Table S2**) has GSEA pathways comprised of chromatin assembly or disassembly, protein kinase binding, kinase binding, negative regulation of cell adhesion, DNA damage and integrity checkpoints. The clade associated with the color green (DEND meta-clade G) has GSEA pathways comprised of endoplasmic reticulum and pathways associated with lipid, glycolipid and alcohol metabolism. Genes associated with the cyan colored sub-clade (DEND meta-clade I) represent the extremes of over expression relative to universal RNA, and expression values much greater when compared to the ASPS-tissue samples. All of these GSEA pathways are related to the cell cycle and include the pathway genes CHK1, RAD50 and RAD51L3. The genes associated with the magenta colored sub-clade (meta-clade J) also comprise genes that are highly over expressed with respect to universal RNA and have higher expression in ASPS-1 when compared to the ASPS-tissue samples. These GSEA pathways include leukocyte chemotaxis and leukocyte migration, transmembrane receptor protein tyrosine kinase activity, transmembrane receptor protein kinase activity, protein tyrosine kinase activity, protein tyrosine kinase activity and KEGG renal cell carcinoma; all containing the pathway genes MET and EPHA5. These latter GSEA pathways suggest the involvement of chemokines and tyrosine kinases, either in the etiology or diagnosis of ASPS [21] or/and as potential therapeutic targets (Phase II Study of Cediranib (AZD2171) in Patients With Alveolar Soft Part Sarcoma, NCI-09-C-0192, NCT00942877).

These findings indicate that genes over expressed in the pooled ASPS-tissue samples relative to ASPS-1, and over expressed with respect to universal RNA, point to pathways related to wound healing that include immune, chemokine and metabolic responses, while genes associated with ASPS-1 and over expressed with respect to universal RNA point to pathways involved in cellular proliferation. Evident from these clustering steps is the association of gene subsets, based only on differential gene expression, that are related to different GSEA pathways. In the next section the analysis will focus on the individual patient data, combined with the RT-PCR measures of the ASPL-TFE3 fusion transcript in these patients.

### SOM Analysis

**Figure 5:**
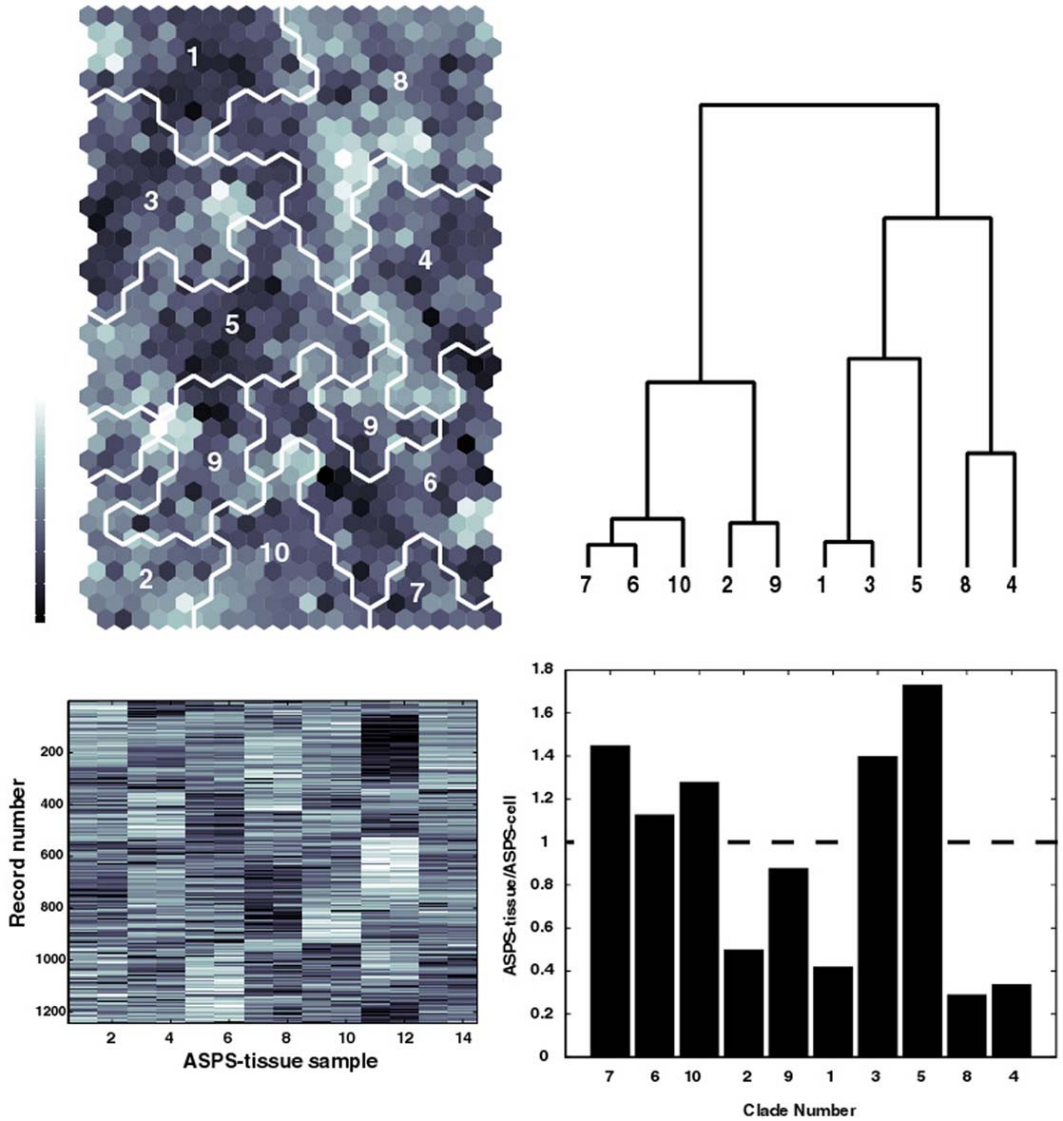
Top left panel displays the SOM for the 1244 individual patient gene expressions from ASPS-tissue and ASPS-1 (1244 genes). SOM colors indicate the similarity of gene measurements between SOM clusters (dark∶ similar light∶less similar). Dendrogram (clipped at 10 SOM meta-clades) from hierarchical clustering of SOM codebook vectors is displayed in the upper right panel. Corresponding SOM meta-clades are identified on the SOM by the white boundary lines. Matching labels appear on the SOM regions and SOM dendrogram. Lower left panel displays the gene expression measurements for the 1244 ASPS-tissue genes for 7 patients, done in replicate. Records are ordered from top to bottom from the left to right meta-clades of the dendrogram. Record boundaries are: SOM meta-clade 7; rows 1–41, SOM meta-clade 6; rows 42–256, SOM meta-clade 10; rows 257–340, SOM meta-clade 2; rows 341–425, SOM meta-clade 9; rows 426–526, SOM meta-clade 1; rows 527–646), SOM meta-clade 3; rows 647–787, SOM meta-clade 5; rows 788–934, SOM meta-clade 8; rows 935–1143, SOM meta-clade 4; rows 1144–1244, Lower right panel displays the histogram for the ratio of counts of genes in the ASPS-tissue set to counts of genes in the ASPS-1 set. Dashed horizontal line defines cases where equal fractions of ASPS-tissue and ASPS-cell genes occur in a meta-clade. Here SOM meta-clades (7, 6, 10, 3 and 5) represent ASPS-tissue genes greater in abundance than found for ASPS-1 genes.

Self-organizing maps (SOMs) represent a powerful tool for analyzing multi-dimensional, noisy data into a form that facilitates visualization of clustering results. The upper left panel in **Figure 5** displays the SOM (row dimension 20 column dimension 12 to yield 240 clusters) generated from individual patient gene expression measurements in ASPS-tissue (1244 genes 7 patients 2 replicates). The SOM has been colored in a gray scale identify the similarity of data assigned to each SOM cluster when compared to its nearest SOM neighbors (dark∶most similar; white∶least similar). To facilitate comparisons of the SOM results with the results derived from the earlier analysis of pooled patient data (ASPS-tissue) versus ASPS-1, all the SOM clusters have been used to generate a SOM meta-clustering of this data. Here the representative cluster for each of the 240 SOM nodes provides input for hierarchical clustering. A dendrogram of the SOM meta-clusters is displayed in the upper right panel of **Figure 5**. This tree has been arbitrarily clipped to display only ten meta-clades. The SOM regions for each meta-clade are displayed as white boundaries in the upper left panel. This result finds, for example, that SOM meta-clades 6, 7 and 10 share similarity in their pattern of gene expressions. Consistent with the SOM dendrogram, SOM meta-clades 6, 7 and 10 are also nearest neighbors. The lower left panel displays the patient gene expression data for these 1244 genes. The patient replicates appear as adjacent columns and provide an indication of the reproducibility of these values. Data from the seven patient tumors is ordered arbitrarily along the x-axis. This gene expression data has been sorted from top to bottom to correspond to the SOM meta-clade groupings appearing in the upper panels. For example, SOM meta-clade 1 and SOM meta-clade 3, adjacent branches in the SOM dendrogram, and SOM neighbors, appear as rows 527–646 and rows 647–787, respectively, in the display of the patient data. The patient data for these genes' expressions display a pattern, as, for example, with column 11–12, corresponding to the 6^th^ patient's measurements, having relatively high gene expressions for rows 527–787, when compared to the other patients' data. In general, this patchwork appearance serves as an illustration of the heterogeneity between patient measurements, and as the basis for dividing these genes into groups, each sharing a similar pattern across the patient samples. This patchwork appearance lends further support to the premise that a linear model may serve to capture patient-specific differences in gene expressions for later comparisons with RT-PCR data.

It is important to emphasize that the 1244 genes used for SOM analysis were derived collectively from the ASPS-tissue (i.e. patient pooled) and ASPS-1 data. Attempts to derive this set of genes by sampling the tails of over and under expressed genes from only the ASPS-tissue data were able to identify many of the *over* expressed genes within this set of 1244, yet few of the *under* expressed genes. Although not pursued in detail, the majority of these under expressed genes appear in the lower left portion of ASPS-tissue versus ASPS-1 scatterplots in **Figure 2**.

The lower right panel in **Figure 5** displays a histogram of the ratio of gene counts from the ASPS-tissue overexpressed genes to the ASPS-1 overexpressed genes, determined for each SOM meta-clade. Values above or below the horizontal dashed line correspond to cases where the ASPS-tissue genes are in greater or lesser abundance, respectively, when compared to the ASPS-1 gene set. The remarkable finding is that the pooled and individual genes can be segregated into groups comprised of similar genes. For example, SOM meta-clades 7, 6, 10, 3 and 5 include patient records abundant in genes over expressed in ASPS-tissue samples when compared to ASPS-1. These SOM meta-clades appear as a diagonal band running midway through the SOM from the 10 o'clock to 4 o'clock position. Conversely, the SOM region corresponding to instances where ASPS-1 gene expressions are greater than ASPS-tissue gene expressions appear above and below this diagonal region. This result provides motivation for subjecting these packets of SOM clustered genes to GSEA analysis, in the same manner as previously used for the pooled patient gene sets versus ASPS-1 gene sets.

Genes in SOM meta-clades most abundant in ASPS-tissue genes (SOM meta-clades 7, 6, 10, 3 and 5) yield the GSEA pathways that, for the most part, recapitulate the results for the pooled ASPS-tissue data. These pathways are dominated by cellular processes involving the extracellular matrix and adhesion. **Table S3** lists these GSEA pathways. Genes in the remaining SOM meta-clades find GSEA pathways consistent with those found for the ASPS-1 data set. These pathways are dominated by cell cycle and mitotic processes. **Table S4** lists these GSEA pathways. Noteworthy in these results is that gene subsets provided for GSEA have not been segregated in any way other than to use the individual patient data as input to SOM clustering. The information contained within the individual patients' gene expressions appears sufficient to identify ASPS-specific pathways similar to those found from the pooled ASPS-tissue versus ASPS-1 data sets.

**Figure 6:**
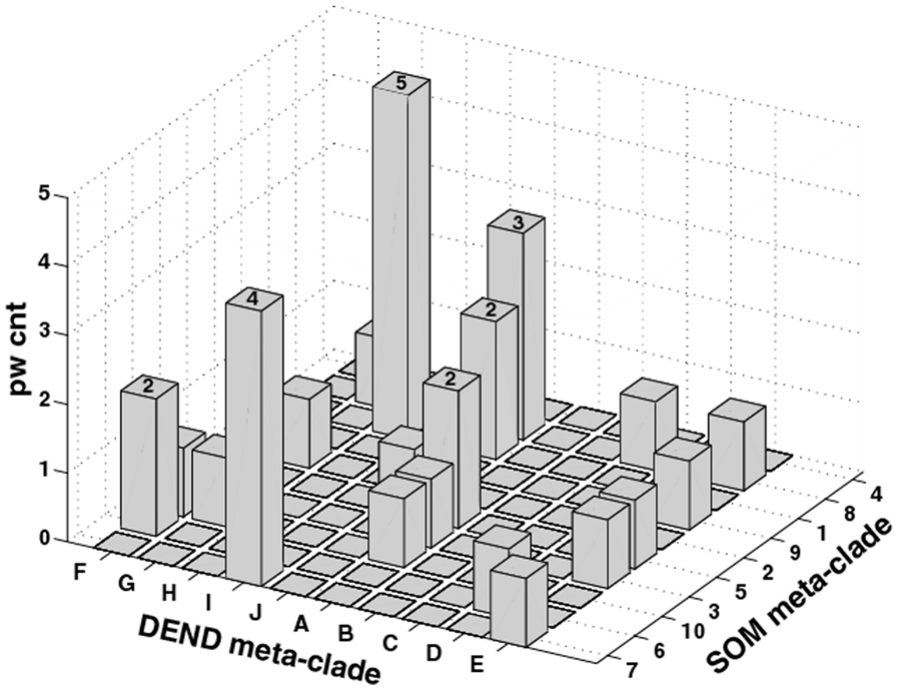
Three-dimensional histogram for count of pathways (pw cnt) shared from the GSEA analysis of gene expressions derived from the patient pooled/ASPS-1 data (axis labels DEND meta-clade, with clade letters A–J) and the individual patient data (axis labeled SOM meta-clades, with meta-clades numbered 1–10). Genes designated with an asterisk are common to pathways identified using the ASPS-tissue: ASPS-1 data set and the individual patient dataset.

The next step in the analysis identifies mutual GSEA pathways determined from the analysis of pooled patient (ASPS-tissue) and cellular gene expression (ASPS-1) data versus the SOM meta-clustering of the individual patient samples. The general premise here is that the mutual appearance of selected pathways derived from these two independent approaches will focus these pathways and their associated pathway gene expressions into ASPS-specific genetic signatures. **Figure 6** displays a 3-dimensional histogram for the co-occurrence of GSEA pathways (number count appears as the Z-axis) in DEND meta-clades A-J (x-axis) and SOM meta-clades 1–10 (y-axis). Twenty-nine GSEA pathways are found to co-exist at least once within the 10 DEND meta-clades and 10 SOM meta-clades. The mutual pathways and their associated gene subsets for these cases are listed in **Table S5**, ordered from largest (n = 5) to smallest (n = 1) counts of mutual pathways. The six instances where more than one common GSEA pathway exists have their number counts at the top of each histogram. The genes listed in the third column of **Table S5** identify the gene subsets defining each GSEA pathway. Asterisks identify pathway genes found mutually from the two different methods of clustering analysis.

These results find that parallel data analyses converge to a mutual set of 29 GSEA pathways and 75 genes that characterize this set of ASPS gene expression measurements. Cell-cycle related processes represent a large share of these pathways, with the finding of a small subset of genes in common to these pathways (denoted with asterisks*), including; BUB1, CCNB1, KIF2C, IL8 and RAB51L3 (cell cycle) and MET, EPHA5, FLT1, NRP2 and ACVR1C (receptor protein kinase pathways). Tumor stromal pathways are also in evidence with the common genes including; TLR4, TLR7, HLA-DRA, HLA-DQA1, CXCR4, FOS and TGFB2 (immune surveillance and chemokine pathways) and NF2, ITGA7, PGF, ITGA4, PDGFD and PARVA (focal adhesion). These results suggest a connection between pathways related to cell survival and pathways related to a supportive stromal environment.

### Incorporation of RT-PCR ASPL-TFE3 Fusion Data

**Figure 7:**
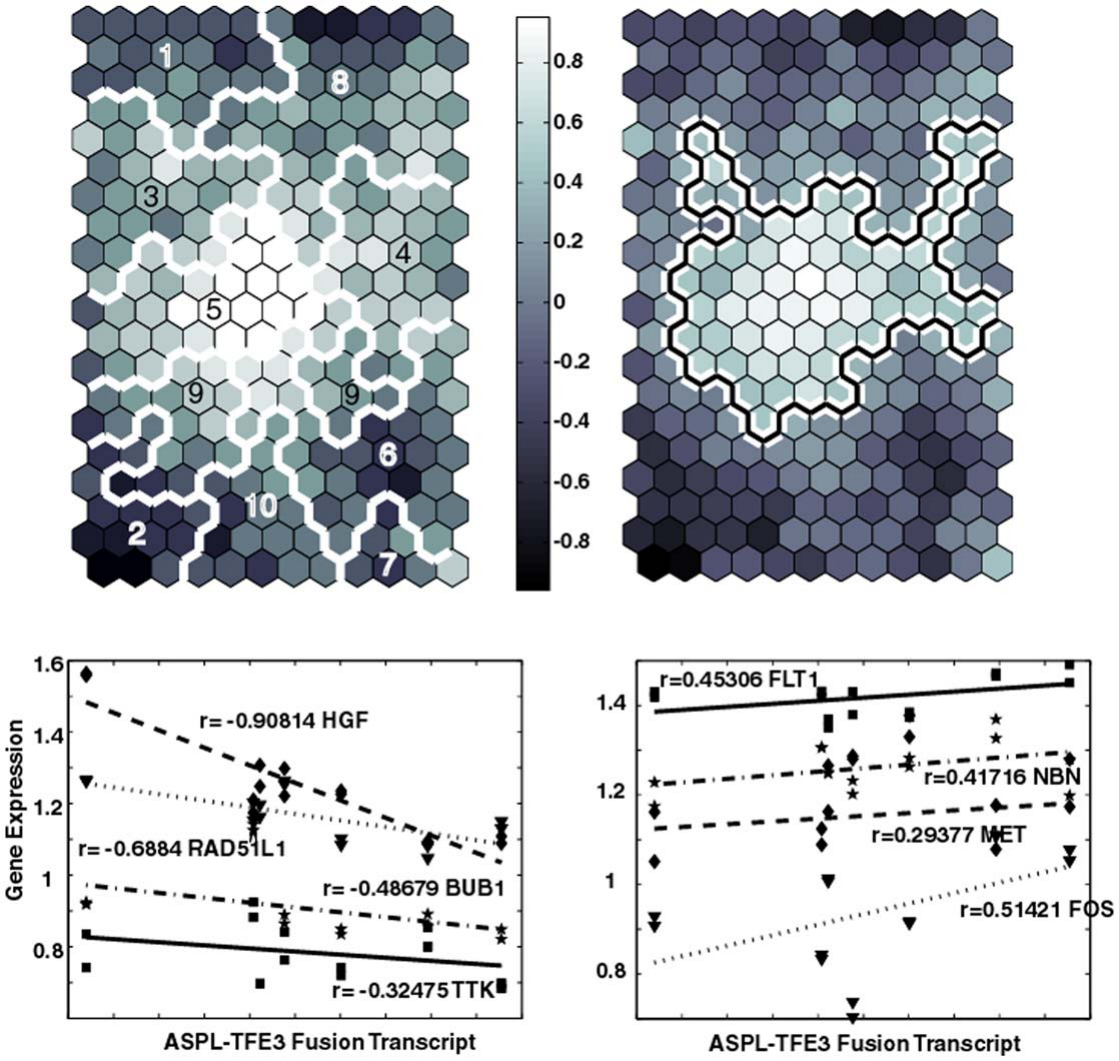
Upper left panel displays SOM (20×12) based on the 1244×14 patient gene measurements. Colors represent the PCCs of the ASPL-TFE3 fusion transcript values against the SOM codebook vectors for each cluster. These codebook vectors represent the best representative of the gene expressions contained within a SOM cluster (typically 5–10 genes). White boundaries correspond to SOM meta-clades. Upper right panel displays the same SOM image with the boundary line (white with black inlay) identifying clusters with the most significant positive PCC values (ASPL-TFE3 fusion transcript versus patient gene expressions). The region scribed by this boundary encompasses most of SOM meta-clade 5, and includes portions of SOM meta-clades 3, 4, 8 and 9. Lower panels plot patient ASPL-TFE3 fusion transcript to gene expressions for selected genes. Lower left and right panels depict examples of negatively and positively correlated genes, respectively. Positive: FLT1:SOM meta-clade 8 MET:SOM meta-clade 9, PGF SOM meta-clade 3, NBN SOM meta-clade 8. Negative: HGF:SOM meta-clade 2, BUB1:SOM meta-clade 1, RAD51L1:SOM meta-clade 7, TTK:SOM meta-clade 1.

The individual patient gene expression data can be analyzed further with RT-PCR determinations of the ASPL-TFE3 fusion transcript. The upper left panel in **Figure 7** displays the 20×12 SOM used earlier to analyze the PCA-derived 1244 differentially expressed genes. The white boundary lines, associated with the previously derived SOM meta-clades, subdivide the patient's gene expression data. The upper right panel in **Figure 7** displays this SOM, with its clusters now colored in a gray scale to indicate the Pearson correlation coefficient (PCC) of the patient's ASPL-TFE3 fusion transcript with patient's gene expressions within each SOM cluster. Here the SOM clusters are colored from most positive (white) to most negative (black) correlations between the average of patient gene expressions within a SOM cluster and the ASPL-TFE3 fusion transcript. The most positively correlated region lies in SOM meta-clade 5 and also corresponds with the SOM regions most associated with the genes over expressed in the ASPS-tissue compared to ASPS-1. This result supports the use of ASPS tissue data as important for identifying ASPS-specific pathways and genes. However, the second most positively correlated SOM region overlaps portions of SOM meta-clades 3, 4 and 8, all of which correspond to SOM regions where genes are over expressed in ASPS-1 versus ASPS-tissue samples. In contrast, the SOM region exhibiting the most negative correlation with the ASPL-TFE3 fusion transcript data lies in SOM meta-clade 2, associated with genes over expressed in the ASPS-tissue versus ASPS-1. The second most negatively correlated SOM region corresponds to SOM meta-clade 1; a region comprised mainly of genes over expressed in the ASPS-1 samples versus ASPS-tissue samples.

Examples of individual correlations are displayed in the lower panels of **Figure 7**; where the left and right plots display negatively and positively correlated genes, respectively. As expected, patient HGF gene expression is strongly negatively correlated with the ASPL-TFE3 fusion transcript. Although not shown here, RAB27A also shares this strong negative correlation; both gene's expression are known to be directly [22] or indirectly [23] under the control of TFE3. FLT1, the fms-related tyrosine kinase 1 (vascular endothelial growth factor/vascular permeability factor receptor) member of the family of receptor tyrosine kinases (RTKs) also displays a strong positive correlation with the ASPL-TFE3 fusion transcript. This positive correlation has been the basis for proposing anti-angiogeneic-based therapeutic strategies [18], [19]. The proto-oncogene MET, also known as hepatocyte growth factor receptor, HGRF, is in the tyrosine kinase family of oncogenes, and also is positively correlated with the ASPL-TFE3 fusion transcript. Both HGF and HGFR have been have been proposed as targets for cancer therapy [24].

The convergent pathways and genes derived from this collective ASPS data set, listed in **Table S5**, can be examined for cases where the SOM meta-clades overlap with SOM meta-clades found to be most correlated with the ASPL-TFE3 fusion transcript data. Pathways extracted from genes associated with SOM meta-clades 3, 4, 5, 8 and 9 are listed in **Table 2**. These ASPS-specific pathways feature tyrosine-kinase activity, immune surveillance and focal adhesion amongst those associated with gene expressions most positively correlated with transcript levels. These pathways, and their associated genes, represent potential ASPS therapeutic targets.

**Table 2:** Convergent pathways where the SOM meta-clades overlap with SOM clades found to be most positively correlated with the ASPL-TFE3 fusion transcript data.

SOM meta-clade	Pathway
8	Protein tyrosine kinase activity
8	Transmembrane receptor protein kinase activity
8	Transmembrane receptor protein tyrosine kinase activity
8	DNA damage response signal transduction by p53 class mediator
5	Cellular morphogenesis during differentiation
5	KEGG intestinal immune network for IgA production
5	KEGG Leishmania infection
3	BIOCARTA cardiac EGF pathway
3	Focal adhesion
9	Focal adhesion

Pathways extracted from genes associated with SOM meta-clades 3, 5, 3 and 9.

**Table 3** lists the results from extracting pathways associated with negative ASPL-TFE3 gene correlations. The isolation of cell-cycle related pathways represents a most unusual result. The pathways associated with SOM meta-clade 1 and 7 are derived from instances where their component genes are all over expressed with respect to universal RNA and have their expression values greatest in ASPS-1, when compared to the ASPS-tissue samples. Apparently the cell cycle control imposed by TFE3 alone is diminished by its fusion transcript, however the genetic components of the cellular machinery needed for proliferation are responding to signals directing cellular mitosis and meiosis. Accordingly, these genes, albeit over expressed with respect to universal RNA, are negatively correlated with the ASPL-TFE3 fusion transcript. Hence most of the necessary components exist to perform cellular proliferation functions, but the transcriptional directive from TFE3 appears to be absent.

**Table 3:** Convergent pathways where the SOM meta-clades overlap with SOM clades found to be most negatively correlated with the ASPL-TFE3 fusion transcript data.

SOM meta-clade	Pathway
1	KEGG cell cycle
1	Mitosis
1	M phase of mitotic cell cycle
1	regulation of cell cycle
1	regulation of mitosis
1	leukocyte chemotaxis
1	leukocyte migration
7	DNA recombination
7	meiosis I
7	meiotic cell cycle
7	meiotic recombination
6	interleukin 8 biosynthetic process
6	negative regulation of cell adhesion
2	alcohol metabolic process
10	BIOCARTA Toll pathway
10	KEGG pantothenate and CoA biosynthesis
10	BIOCARTA ASHP pathway

Pathways are extracted from genes associated with SOM meta-clades 1, 7, 6, 2, 10.

## Discussion

**Figure 8:**
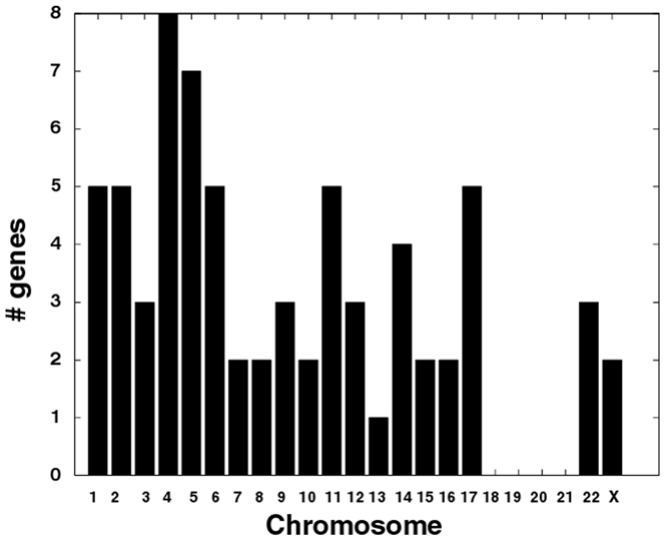
Bar chart for number of genes per chromosome. Genes are selected from **Table S5** (n = 75 genes).

Collective analysis of patient ASPS and ASPS-1 gene expressions and the patient-derived ASPL-TFE3 fusion transcript yielded a consistent genetic picture of ASPS-specific pathways and their associated genes. The cellular processes affected by the non-reciprocal t(X;17) chromosomal translocation, that can be collectively linked to measures of gene expression derived independently from ASPS patient tissue and an ASPS cell, identify aberrant regulation of the cell-cycle and tissue stroma-related adhesion pathways as ASPS genetic signatures. These pathways largely reflect the nature of what is known about these tumor cells and their behavior. Relating these pathways to the biology of ASPS is an essential step towards understanding their roles in this disease and proposing therapeutic strategies. An obvious first step is to explore the occurrence of the 75 genes listed in **Table S5** according to their chromosomal origins. **Figure 8** displays a bar chart for the count of these genes according to chromosome. Noteworthy in this plot is the existence of 5 genes associated with chromosome 17 and 2 genes with chromosome X (**Table 4**). The genes within the same cytogenetic band as TFE3 (chr17q25) include ARHGDIA and BIRC5, functioning, respectively, in pathways related to cell adhesion and cell cycle. Consistent with the observed negative correlation between TFE3 regulated gene expressions and ASPL-TFE3 transcript levels, this analysis identifies genes such as HGF, ARHGDIA and BIRC5, albeit at a significance level of p<0.1. The appearance of BIRC5 on cytogenetic band 17q25 and its negative correlation between ASPL-TFE3 fusion transcript levels and patients' gene expression, clearly points to an important role of the cell cycle in ASPS, either directly through BIRC5, or more generally through other genes involved in the cell cycle. **Table 4** and **Table S5** both include the GSEA cell cycle pathways of meiosis, meiotic cell cycle, meiotic recombination and DNA recombination and the genes CHK1, RAD50, RAD51L3 and RAD50L1. Amongst this list of genes is the appearance of CHK1, which encodes a protein kinase required for DNA damage checkpoint control; also found to be over expressed in ASPS-1 relative to ASPS tissue. This checkpoint control may be linked to the earlier described elevation in gene expression for cell cycle genes, and to the arrested growth rate of ASPS tumor cells. Recently CHK1 inhibitors have been proposed as cancer therapeutics on the basis of their ability to activate cell cycle checkpoints in p53 defective cancer cells [25]. Others have proposed that the elevated levels of CHK1 in some cancers, postulated to result from reduced capacity for its degradation, could provide a selective advantage to cancer cells by conferring chemo-resistance [26]. Ma et al. [25] and Reed and Altiok [27] propose that CHK1 inhibition may release its checkpoint function, thereby sensitizing tumor cells to anticancer agents. This possibility, combined with the current analyses' emphasis on the role of cell cycle in ASPS, warrants further investigation for CHK1 inhibitors in the treatment of these chemo-resistant ASPS tumor cells.

**Table 4:** Details for genes associated with Chromosomes 17 and X.

Gene	Cytoband	GSEA p-value	Gene Description	GSEA Pathway Description
ARHGDIA	chr17q25	5.53e-4	Rho GDP dissociation inhibitor (GDI) alpha	Negative regulation of cell adhesion
BIRC5	chr17q25	5.53e-4	baculoviral IAP repeat-containing 5 (survivin)	Kegg cell cycle, mitosis, M-phase of mitotic cycle, regulation of cell cycle
CCL4	chr17q21	1.34e-3	chemokine (C-C motif) ligand 4	Protein tyrosine kinase activity
CDC6	chr17q21	1.34e-3	CDC6 cell division cycle 6 homolog (S. cerevisiae)	Kegg cell cycle
RAD51L3	chr17q11		RAD51-like 3 (S. cerevisiae)	Meiosis_I, meiotic cell cycle
KAL1	chrxp22	4.41e-5	Kallmann syndrome 1 sequence	Cellular morphogenesis during differentiation
TLR7	chrxp22	4.41e-5	Toll-like receptor 7	Interleukin 8 biosynthetic process

Columns include a) HUGO name, b) chromosome position, c) GSEA probability for genes in same cytoband, d) brief description of gene, and e) GSEA pathway.

Other interesting genes implicated in these results are CCL4 and CDC6, located in the TFE3 neighboring cytogenetic band, chr17q21, with cellular functions related to protein tyrosine kinase activity and cell cycle. CCL4, also known as macrophage inflammatory protein 1-β, is a regulator of macrophage migration and signals through the G-protein coupled beta chemokine receptor, CCR5. Protein kinases linked to this chemokine family include the SRC kinase Lyn, PI3K, focal adhesion related kinase Pyk2, and members of the MAPK family. siRNA gene silencing demonstrated that chemotaxis requires activation of Pyk2, PI3K p85, and Lyn, as well as MAPK ERK. MIP-1β activation of CCR5 triggered translocation of Pyk2 and PI3K p85 from the cytoplasm to co-localize with Lyn, at the plasma membrane, with formation of a multi-molecular complex. In addition, arrestins, also recruited to CCR5, impaired complex formation and macrophage chemotaxis toward MIP-1β when down-regulated. Together, these results identify a novel mechanism of chemokine receptor regulation of chemotaxis linking CCR5 to multiple downstream signaling molecules [28].

Localization of CCL4 to chromosome 17q21, raises a number of interesting possibilities for future experiments. CCL4 is also a member of the family of beta chemokines, and functions in pathways associated with protein tyrosine kinase activity (see **Table 4**). Recently, beta chemokine CCL5 neutralization has been found to restrict cancer cell growth in colorectal cancer [29]. This study explored tumor-stromal communications that favor tumor development by signaling growth factors, angiogenesis, modulation of the ECM, and recruitment of additional stromal cells as part of the immune evasion mechanisms of cancer. Colon cancer promoters, which also correlate with tumor grade and shorter patient survival, include VEGF, FGF and PDGF. Increasing evidence supports a role of chemokines produced within the tumor microenvironment in tumor pathogenesis [30]–[33]. One study reports clinical efficacy when treating an ASPS patient with interferon alpha 2a [34]; an effect possibly mediated by the role of interferons in angiogenesis [35] or by the capacity of interferons to activate specific signaling cascades involving chemokines [36].

CCL4 is also a member of the family of beta chemokines that may function in ASPS in ways similar to those observed for CCL5 in colon cancer. Recent studies find activation of phosphoinositide 3-kinases by the CCR4 ligand [37], a result similar to that reported above for CCL5. A comparison of CCL5 patient expression with ASPL-TFE3 fusion transcript levels finds a strong positive correlation (r = 0.78 p = 0.0036), placing it above the observed positive correlation for FLT1. CDC6, also on chr17q21, has expression values positively correlated with ASPL-TFE3 fusion transcript, albeit with a significance value above the standard threshold (r = 0.19, p = 0.12). RAD51L3, also located in chr17q21, functions in pathways related to cellular meiosis and exhibits a strong negative correlation with the ASPL-TFE3 fusion transcript. Collectively these results can be used to hypothesize experimental modulation of pathways associated with genes found on chromosome 17 as a means to identify potential therapeutic targets in the treatment of ASPS.

Many of the results presented here are consistent with recent reports describing targeted therapies for the treatment of soft tissue sarcomas [38], [39]. Stacchiotti *et al.* [40] found direct evidence for an antitumor effect from treatment of advanced ASPS with sunitinib. Their antiproliferative and biochemical assays found sunitinib to markedly impair ASPS cell growth and switch off PDGFBR. The results presented here (**Table S5**) find a role for platelet-derived growth factor proteins in focal adhesion pathways. Their study [40] further confirmed a physical association between PDGFBR targets in ASPS cells, as well as MET-ligand dependent activation. A recent report [41] finds that the MET inhibitor ARQ197 yielded a response in a phase II study of clear cell sarcoma. These examples, and others (see the review by Taylor et al. [42]) are consistent with the findings here of the importance of protein tyrosine kinase pathways, and in particular the role of MET, as ASPS-specific pathways and an ASPS-specific gene, respectively (**Table S5**). Early studies found an association between certain sarcomas and insulin growth factor (IGF) signaling [43], [44]. Recent developments now find that IGF and insulin receptors facilitate sufficient cross-talk between various pathways to consider them as important anticancer targets. In support of this claim, a mechanism has been proposed linking the translocation associated with Ewings sarcoma with the IGF binding protein 3 (IGFBP3) promoter, reducing IGFBP3 production and effectively up-regulating IFG1 [45]–[47]. The results reported here (**Table S5**) implicate IGF1 as an ASPS-specific target with an important role in focal adhesion pathways. Finally, the earlier mention here of cediranib (see section *GSEA Results for clustered subsets of pooled ASPS-tissue and ASPS-1 genes:*), can now be updated with the finding of numerous objective remissions, apparently related to its role as a protein tyrosine kinase inhibitor of VEGF and PDGFR [48]. While the reported role of cediranib appears to involve inhibition of angiogenesis, the analysis here points to an additional role in focal adhesion as well as the possibility of interaction with chemokines that affect tyrosine kinase activity.

In summary, the results from bioinformatics mining of the collective ASPS data raise a number of testable hypotheses regarding a limited set of cellular pathways as potential therapeutic targets in the treatment of ASPS. The linear model introduced here to detect important genetic signals is generally applicable to instances where heterogeneous tissue samples are used for gene expression profiling.

## Supporting Information

Figure S1 **GSEA output for genes (y-axis) and pathways (x-axis).** Black boxes indicate occurrence of 2 or more genes in a GSEA pathway. GSEA results are derived from the 542 filtered genes above the 1^st^ PC (See Figure 2). These genes are over expressed in ASPS-tissue relative to ASPS-1.
(DOC)

Figure S2 **GSEA output for genes (y-axis) and pathways (x-axis).** Black boxes indicate occurrence of 2 or more genes in a GSEA pathway. GSEA results are derived from the 702 filtered genes below the 1st PC (See Figure 2). These genes are over expressed in ASPS-1 relative to ASPS-tissue.
(DOC)

Table S1 GSEA Pathways and Pathway Genes for the DEND meta-clades derived from the clustering of ASPS-tissue genes (See **Figure 3**).
(DOC)

Table S2 GSEA Pathways and Pathway Genes for the DEND meta-clades derived from the clustering of ASPS-1 genes (See **Figure 4**).
(DOC)

Table S3 GSEA pathways for genes associated with SOM meta-clades 7, 6, 10, 3 and 5.
(DOC)

Table S4 GSEA pathways for genes associated with SOM meta-clades 2,9,1,8 and 4.
(DOC)

Table S5 Results for convergent pathways derived from the pooled ASPS-1 and ASPS-tissue analysis and the SOM-based analysis of individual patient gene expressions. Column 1 lists the meta-clade identifiers (DEND meta-clade: SOM meta-clade), column 2 lists the GSEA pathways and column 3 lists the pathway genes identified from each analysis.
(DOC)
