# Supplementary material for: Bioinformatic Analysis of Patient-Derived ASPS Gene Expressions and ASPL-TFE3 Fusion Transcript Levels Identify Potential Therapeutic Targets
Source: PLoS One. 2012 Nov 30;7(11):e48023. doi: 10.1371/journal.pone.0048023 (PMC3511488; doi:10.1371/journal.pone.0048023)
Supplement: Table S1 — GSEA Pathways and Pathway Genes for the DEND meta-clades derived from the clustering of ASPS-tissue genes (See Figure 3 ). (DOC) [file pone.0048023.s003.doc]

| GSEA Pathway | Pathway Genes |
| --- | --- |
| black: DEND meta-clade A |  |
| extracellular region, GO:0005576 | SLIT2, KAL1, KL, LYZ, INHBB, COL4A5, COL6A3, FBLN5, SPINT2, FRZB, and FXYD6 |
| Biocarta ASHP pathway, | HBA1 and HBA2 |
| KEGG viral myocarditis | HLA-DRA, HLA-DQA1, HLA-DQB1, and MYH11 |
| green: DEND meta-clade B |  |
| enzyme inhibitory activity, GO:0004857, and enzyme regulatory activity, GO:0030234, | AGT, OAZ3, and BCR, |
| membrane, GO:0005624, and cell fractions, GO:0000000267 | OLR1, SLC12A6, and LASS5 |
| cytosol, GO:0005829 | HBB and ACY1 |
| red: DEND meta-clade C |  |
| positive regulation of cell proliferation, GO:0008284, and regulation of cell proliferation, GO:0042127 | FLT1, NRP1, PTPRC, CXCL10, CD86, HCLS1, and IGF1, |
| extracellular region, GO:0005576 | CCL4, RNASE6, C1Qa, C1QB, THBS4, COL5A1, ECM2, CD248, FGL2, and F13A1, |
| chemokine activity, GO:0008009, immune response, GO:0006955, inflammatory response, GO:0006954, defense response, GO:0006952, | CXCL10, CCL4 and CCL3. |
| cyan: DEND meta-clade D |  |
| sarcomere, GO:0030017, myofibril, GO:0030016, contractile fiber part, GO:0044449, contractile fiber, GO:0043292, and structural constituent of muscle, GO:0008397 | TTN and NEB, |
| heparin binding, GO:0008201, carbohydrate binding, GO:0030246, and glycosaminoglycan binding, GO:0005539 | POSTN and MDK. |
| magenta: DEND meta-clade E |  |
| KEGG focal adhesion | FLT1, HGF, ITGA1, ITGB5, PDGFD and PARVA |
| basolateral membrane, GO:0016323 | EVL, DST and SLC16A10. |
