# Supplementary material for: Bioinformatic Analysis of Patient-Derived ASPS Gene Expressions and ASPL-TFE3 Fusion Transcript Levels Identify Potential Therapeutic Targets
Source: PLoS One. 2012 Nov 30;7(11):e48023. doi: 10.1371/journal.pone.0048023 (PMC3511488; doi:10.1371/journal.pone.0048023)
Supplement: Table S2 — GSEA Pathways and Pathway Genes for the DEND meta-clades derived from the clustering of ASPS-1 genes (See Figure 4 ). (DOC) [file pone.0048023.s004.doc]

| black: DEND meta-clade F |  |
| --- | --- |
| chromatin assembly or disassembly GO:0006333 | HELLS, TLK1, SMARCE1 and HDAC8 |
| protein kinase binding, GO:0019901, kinase binding, GO:0019900, and negative regulation of cell adhesion, GO:0007162 | CDKN2A, ADAM10, TRIB3, PTPRR, SQSTM1 and PDLIM5 |
| DNA damage checkpoint, GO: 0000077, DNA integrity checkpoint, GO:0031570 | CHK1, HUS1 and CCNA2 |
| regulation of myleoid cell differentiation, GO:0045637 | ETS1, SCIN and ZNF675 |
| interleukin 8 biosynthetic process, GO: 0042228 | TLR7 and TLR4, |
| Biocarta P38 MAPK signaling | RIPK1, STAT1 and PLA2G4A |
| green: DEND meta-clade G |  |
| endoplasmic reticulum, GO:0005783 | CLN6, GLA, HMOX1, PLOD2, STS, IDS, ERO1L, YKT6, CTSZ and RTN4R |
| GSEA pathways associated with lipid, glycolipid and alcohol metabolism | CLN6,PPARGC1A,GFPT2,COQ2,IPPK |
| cyan: DEND meta-clade I |  |
| regulation of mitosis(GO:0007088), M-phase(GO:0000279), Mitosis(GO:0007067), M phase of mitotic cell cycle(GO:0000087), cell cycle phase(GO:0022403), spindle(GO:0005819), regulation of cell cycle(GO:0051726), cell cycle(GO:0007049), KEGG cell cycle and cell cycle process(GO:0022402) | CHK1, RAD50 and RAD51L3 |
| magenta: DEND meta-clade J |  |
| leukocyte chemotaxis(GO:0030595) and leukocyte migration(GO:0050900) | DOCK2 and TGFB2 |
| transmembrane receptor protein tyrosine kinase activity(GO:0004714), transmembrane receptor protein kinase activity(GO:0019199), protein tyrosine kinase activity(GO:0032403), protein tyrosine kinase activity(GO:0004713) and KEGG renal cell carcinoma | MET and EPHA5 |
