# Supplementary material for: Bioinformatic Analysis of Patient-Derived ASPS Gene Expressions and ASPL-TFE3 Fusion Transcript Levels Identify Potential Therapeutic Targets
Source: PLoS One. 2012 Nov 30;7(11):e48023. doi: 10.1371/journal.pone.0048023 (PMC3511488; doi:10.1371/journal.pone.0048023)
Supplement: Table S3 — GSEA pathways for genes associated with SOM meta-clades 7, 6, 10, 3 and 5. (DOC) [file pone.0048023.s005.doc]

| General Pathway Description | GSEA Pathway |
| --- | --- |
| Negative regulation of cell adhesion | GO:0007162. Any process that stops, prevents or reduces the frequency, rate or extent of cell adhesion. |
| KEGG ECM receptor interaction | ECM-receptor interaction |
| Cell matrix adhesion | GO:0007160. The binding of a cell to the extracellular matrix via adhesion molecules. |
| Basolateral plasma membrane | GO:0016323. Part of the plasma membrane that includes the basal end and sides of the cell. |
| Cell matrix junction | GO:0030055. A specialized region of connection between a cell and the extracellular matrix. |
| Proteinaceous extracellular matrix | GO:0005578. A layer consisting mainly of proteins (especially collagen) and glycosaminoglycans (mostly as proteoglycans) that forms a sheet underlying or overlying cells such as endothelial and epithelial cells. |
| Cell matrix adhesion | GO:0007160. The binding of a cell to the extracellular matrix via adhesion molecules. |
| Extracellular matrix | GO:0031012. A structure lying external to one or more cells. |
| KEGG intestinal immune network for IgA production | Intestinal immune network for IgA production |
|  |  |
