# Supplementary material for: Bioinformatic Analysis of Patient-Derived ASPS Gene Expressions and ASPL-TFE3 Fusion Transcript Levels Identify Potential Therapeutic Targets
Source: PLoS One. 2012 Nov 30;7(11):e48023. doi: 10.1371/journal.pone.0048023 (PMC3511488; doi:10.1371/journal.pone.0048023)
Supplement: Table S4 — GSEA pathways for genes associated with SOM meta-clades 2,9,1,8 and 4. (DOC) [file pone.0048023.s006.doc]

| General Pathway Description | GSEA Pathway |
| --- | --- |
| M phase | GO:0000279. Progression through M phase of cell division |
| M phase of mitotic cell cycle | GO:0000087. Progression through M phase. |
| Regulation of mitosis | GO:0007088. Any process that modulates the frequency, rate or extent of mitosis. |
| KEGG cell cycle | Cell cycle |
| Positive regulation of cell cycle | GO:0045787. Any process that activates or increases the rate or extent of progression through the cell cycle. |
| Mitosis | GO:0007067. Progression through mitosis. |
| Cell cycle checkpoint | GO:0000075. A point in the eukaryotic cell cycle where progress through the cycle. |
