# Supplementary material for: Bioinformatic Analysis of Patient-Derived ASPS Gene Expressions and ASPL-TFE3 Fusion Transcript Levels Identify Potential Therapeutic Targets
Source: PLoS One. 2012 Nov 30;7(11):e48023. doi: 10.1371/journal.pone.0048023 (PMC3511488; doi:10.1371/journal.pone.0048023)
Supplement: Table S5 — Results for convergent pathways derived from the pooled ASPS-1 and ASPS-tissue analysis and the SOM-based analysis of individual patient gene expressions. Column 1 lists the meta-clade identifiers (DEND meta-clade: SOM meta-clade), column 2 lists the GSEA pathways and column 3 lists the pathway genes identified from each analysis. (DOC) [file pone.0048023.s007.doc]

| DEND:SOM meta-clade pairs | GSEA Pathway | Genes |
| --- | --- | --- |
|  |  |  |
| H:1 | KEGG cell cycle | BUB1*,TTK,CDC23,CCNB1*,MCM4,TGFB2,CDC6,CCNA2 |
| H:1 | mitosis | BUB1*,TTK,BIRC5,CDC23,KIF2C*,CCNA2,NUSAP1 |
| H:1 | M phase of mitotic cycle | BUB1*,TTK,BIRC5,CDC23,KIF2C*,NUSAP1 |
| H:1 | regulation of cell cycle | BUB1*,TTK,BIRC5,CDC23,IL8*,MLF1,TGFB2,CCNA2,NUSAP1,CDC6 |
| H:1 | regulation of mitosis | BUB1*,TTK,BIRC5,CDC23,CCNA2,NUSAP1 |
|  |  |  |
| I:7 | DNA recombination | CHK1,RAD50,RAD51L3*,RAD51L1 |
| I:7 | meiosis I | CHK1,RAD50,RAD51L3*,RAD51L1 |
| I:7 | meiotic cell cycle | CHK1,RAD50,RAD51L3*,RAD51L1 |
| I:7 | meiotic recombination | CHK1,RAD50,RAD51L3* |
|  |  |  |
| J:8 | protein tyrosine kinas activity | MET*,NRP2,EPHA5*,FLT1,TTK |
| J:8 | transmembrane receptor protein kinase activity | MET*,NRP2,EPHA5*,FLT1,ACVR1C |
|  |  |  |
| F:6 | interleukin 8 biosynthetic process | TLR4*,TLR7* |
| F:6 | negative regulation of cell adhesion | CDKN2A,ADAM10,NF2*,ARHGHIB,ARHGDIA |
|  |  |  |
| A:5 | KEGG intestinal immune network for IgA production | HLA-DRA*,HLA-DQA1*,HLA-DQB1,CXCR4*,ITGA4,CD86 |
| A:5 | KEGG Leishmania infection | HLA-DRA*,HLA-DQA1*,HLA-DQB1,FOS*,ITGA4,HLA-DRA,FCGR2A |
|  |  |  |
| J:1 | leukocyte chemotaxis | DOCK2,TGFB2*,IL8 |
| J:1 | leukocyte migration | DOCK2,TGFB2*,IL8 |
|  |  |  |
| G:2 | alcohol metabolic process | CLN6*,PPARGC1A,GFPT2,COQ2*,IPPK |
|  |  |  |
| F:10 | BIOCARTA Toll pathway | TLP4,TLR7,PPARA*,TIRAP* |
|  |  |  |
| G:10 | KEGG pantothenate and CoA biosynthesis | BCAT1*,PANK3,VNN1 |
|  |  |  |
| A:10 | BIOCARTA ASHP pathway | HBA1*,HBA2* |
|  |  |  |
| A:3 | BIOCARTA cardiac EGF pathway | ADAM12,AGT,EDNRA*,FOS |
|  |  |  |
| E:3 | KEGG focal adhesion | FLT1,HGF,ITGA7*,ITGB5,PDGFD,PGF*,PARVA |
|  |  |  |
| J:5 | KEGG Leishmania infection | ITGA4*,HLA-DRA,HLA-DQA1,FOS,FCGR2A |
|  |  |  |
| E:5 | cellular morphogenesis during differentiation | KAL1,NRP1,SPON2*,THY1,CEP290,NRP2 |
|  |  |  |
| E:9 | KEGG focal adhesion | PDGFC,PDGFD*,IGF1,MET,SOS2,PTK2,PARVA*,PARVB,FLT1,HGF,ITGA7,ITGB5,PGF |
|  |  |  |
| E:8 | transmembrane receptor protein kinase activity | MET,NRP2*,EPHA5,FLT1*,ACVR1C* |
|  |  |  |
| C:8 | protein tyrosine kinase activity | FLT1*,NRP1,CCL4,CSF1R |
|  |  |  |
| G:8 | DNA damage response signal transduction by p53 class mediator | IFI16*,NBN* |
