# Supplementary material for: Bioinformatic Analysis of Patient-Derived ASPS Gene Expressions and ASPL-TFE3 Fusion Transcript Levels Identify Potential Therapeutic Targets
Source: PLoS One. 2012 Nov 30;7(11):e48023. doi: 10.1371/journal.pone.0048023 (PMC3511488; doi:10.1371/journal.pone.0048023)
Supplement: Supplementary file 6 [file pone.0048023.s001.doc]

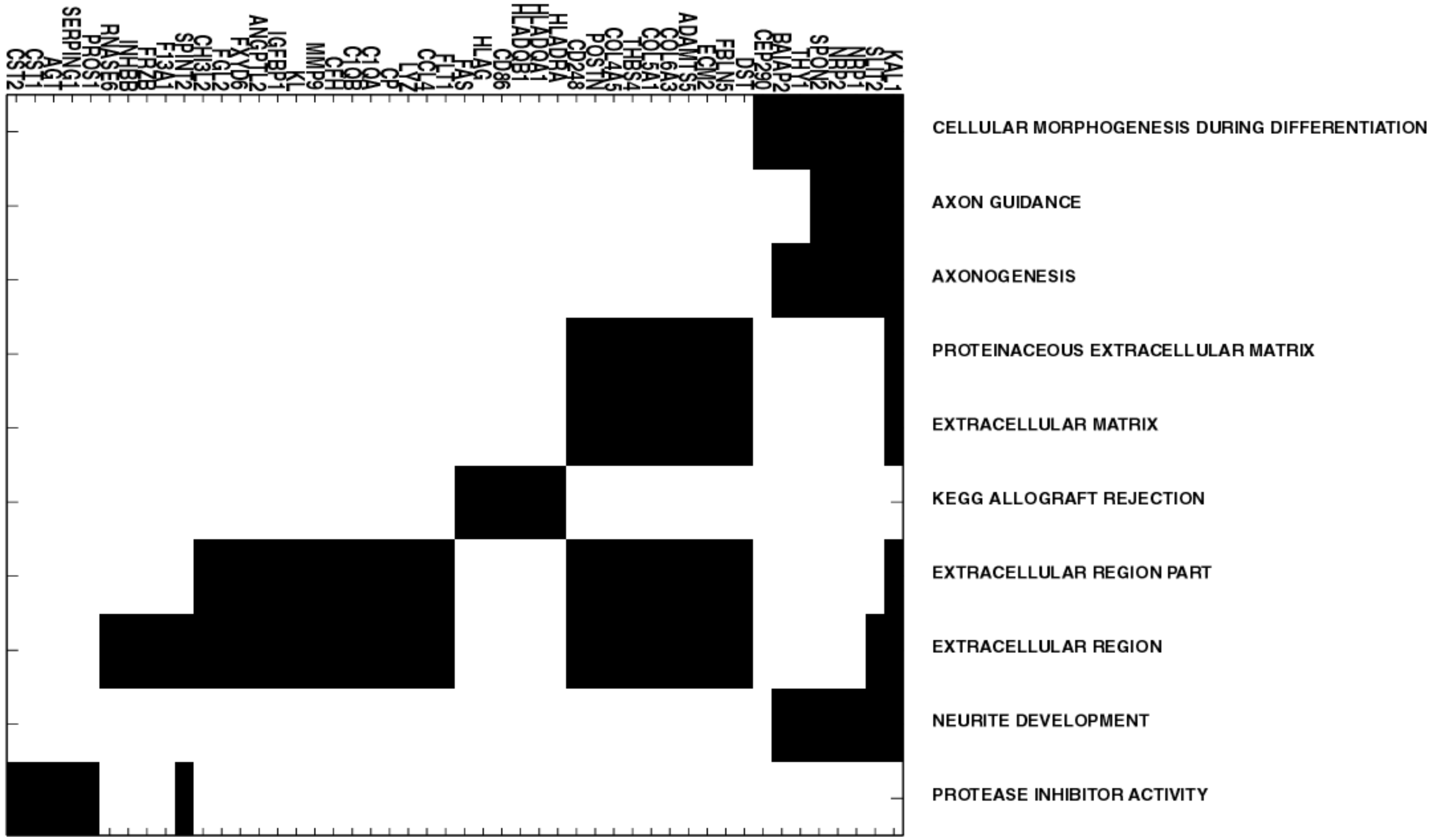

CELLULAR MORPHOGENESIS DURING DIFFERENTIATION
AXON GUIDANCE
AXONOGENESIS
PROTEINACEOUS EXTRACELLULAR MATRIX
EXTRACELLULAR MATRIX
KEGG ALLOGRAFT REJECTION
EXTRACELLULAR REGION PART
EXTRACELLULAR REGION
NEURITE DEVELOPMENT
PROTEASE INHIBITOR ACTIVITY
KAL1
SLIT2
NRP1
NRP2
SPON2
THY1
BAIAP2
CEP290
DST
FBLN5
ECM2
ADAMTS5
COL6A3
COL5A1
THBS4
COL4A5
POSTN
CD248
HLADRA
HLADQA1
HLADQB1
CD86
HLAG
FAS
FLT1
CCL4
LYZ
CP
C1QA
C1QB
CFH
MMP9
KL
IGFBP1
ANGPTL2
FXYD6
FGL2
CHI3L2
SPINT2
F13A1
FRZB
INHBB
RNASE6
PROS1
SERPING1
AGT
CST1
CST2
