# Supplementary material for: Bioinformatic Analysis of Patient-Derived ASPS Gene Expressions and ASPL-TFE3 Fusion Transcript Levels Identify Potential Therapeutic Targets
Source: PLoS One. 2012 Nov 30;7(11):e48023. doi: 10.1371/journal.pone.0048023 (PMC3511488; doi:10.1371/journal.pone.0048023)
Supplement: Supplementary file 7 [file pone.0048023.s002.doc]

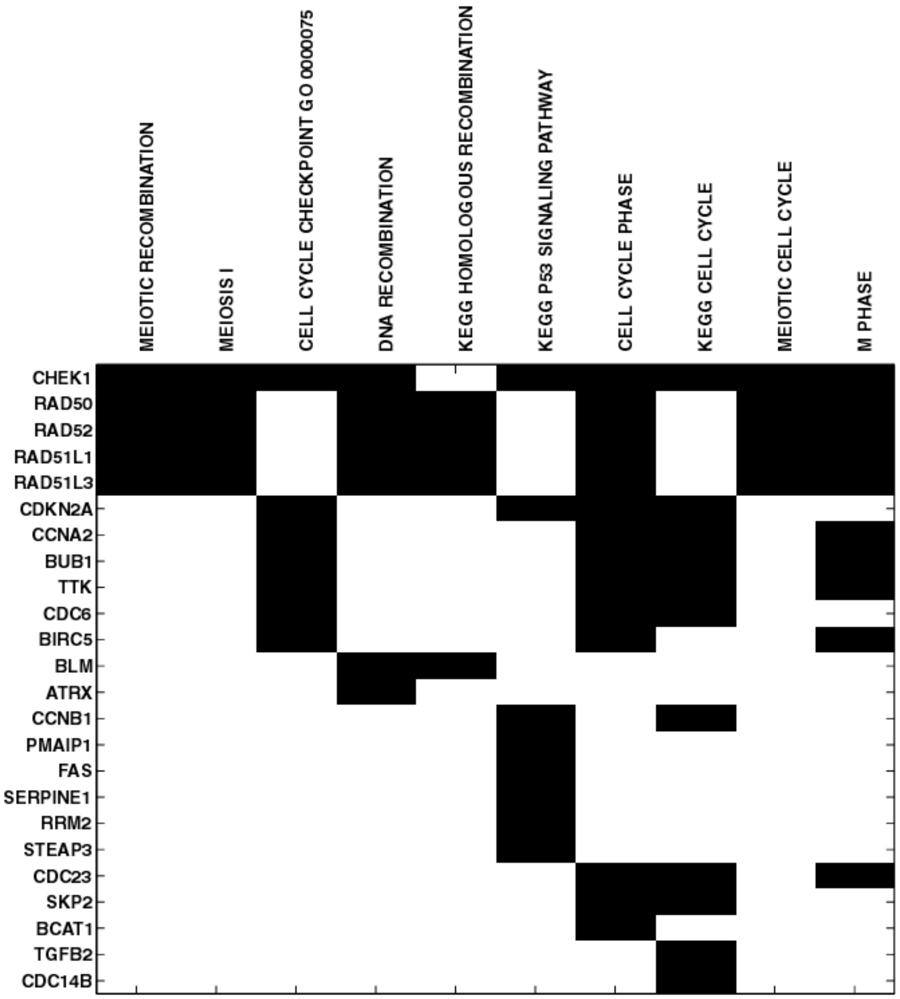

MEIOTIC RECOMBINATION
MEIOSIS I
CELL CYCLE CHECKPOINT GO 0000075
DNA RECOMBINATION
KEGG HOMOLOGOUS RECOMBINATION
KEGG P53 SIGNALING PATHWAY
CELL CYCLE PHASE
KEGG CELL CYCLE
MEIOTIC CELL CYCLE
M PHASE
CHEK1
RAD50
RAD52
RAD51L1
RAD51L3
CDKN2A
CCNA2
BUB1
TTK
CDC6
BIRC5
BLM
ATRX
CCNB1
PMAIP1
FAS
SERPINE1
RRM2
STEAP3
CDC23
SKP2
BCAT1
TGFB2
CDC14B
